# The Western Australian preterm birth prevention initiative: a whole of state singleton pregnancy cohort study showing the need to embrace alternative models of care for Aboriginal women

**DOI:** 10.1186/s12884-022-05222-9

**Published:** 2023-01-04

**Authors:** Ye’elah E. Berman, John P. Newnham, Scott W. White, Kiarna Brown, Dorota A. Doherty

**Affiliations:** 1 Division of Obstetrics and Gynaecology, University of Western Australia, King Edward Memorial Hospital, 374 Bagot Road, Subiaco, Perth, WA 6008 Australia; 2 Department of Obstetrics and Gynaecology, Royal Darwin Hospital, Rocklands Drive, Tiwi, Darwin, NT 0811 Australia; 3 Menzies School of Health Research, John Matthews Building, Corner of Nightingale and Paracelsus Road, Tiwi, Darwin, NT 0810 Australia

**Keywords:** Preterm birth, Prevention, Pregnancy, Aboriginal, Population-based study

## Abstract

**Background:**

Preterm birth (PTB) is the greatest cause of mortality and morbidity in children up to five years of age globally. The Western Australian (WA) PTB Prevention Initiative, the world’s first whole-of-population whole-of-state program aimed at PTB prevention, was implemented across WA in 2014.

**Methods:**

We conducted a prospective population-based cohort study using pregnancy data for singleton births in WA from 2009 to 2019. Logistic regression using the last full year before the Initiative (2013) as the reference, and run charts were used to examine changes in PTB rates compared to pre-Initiative levels, by gestational age group, hospital type, low and high risk of PTB in mid-pregnancy, and onset of labour (spontaneous/medically initiated). Analyses were stratified by Aboriginal and non-Aboriginal maternal ethnicity.

**Results:**

Amongst non-Aboriginal women, there was initially a reduction in the PTB rate across the state, and in recent years it returned to pre-Initiative levels. Amongst Aboriginal women there was a small, non- significant reduction in the state-wide PTB rate in the first three years of the Initiative, followed by a rise in recent years. For non-Aboriginal women, the reduction in the rate of PTB at the tertiary centre was sustained and improved further for women of all risk levels and onsets of labour. This reduction was not observed for Aboriginal women giving birth at the tertiary centre, amongst whom there was an increase in the PTB rate overall and in all subgroups, with the exception of medically initiated PTB. Amongst Aboriginal women the PTB rate has also increased across the state. At non-tertiary hospitals there was a large increase in PTB amongst both Aboriginal and non-Aboriginal women, largely driven by medically initiated late PTB. Maternal risk factors cannot account for this increase.

**Conclusions:**

The reduction in PTB rates amongst non-Aboriginal women at the state’s tertiary hospital demonstrates that with the right strategies, PTB can be reduced. A sustained collaborative model is required to realise this success in non-tertiary hospitals. The series of interventions was of limited use in Aboriginal women, and future efforts will need to be directed at strategies more likely to be successful, such as midwifery continuity of care models, with Aboriginal representation in the healthcare workforce.

**Supplementary Information:**

The online version contains supplementary material available at 10.1186/s12884-022-05222-9.

## Background

Preterm birth (PTB), defined as birth from 20 and before 37 completed weeks of pregnancy is the greatest cause of mortality and morbidity in children up to five years of age globally [1] and is increasing in high income countries [2], including Australia [3]. One in eleven Australians is born too early and among Aboriginal and/or Torres Strait Islander women (hereafter respectfully referred to as Aboriginal) the rate is almost double [3]. Perinatal outcomes are considerably worse for Australian Aboriginal infants, who experience higher rates of PTB, low birthweight and infant mortality both nationally [3], and in Western Australia (WA) [4–6]. The causes of these inequalities are complex and risk factors for poor perinatal outcomes are not necessarily the same in Aboriginal women as in the broader WA obstetric population.

The WA PTB Prevention Initiative (the Initiative) was implemented across WA in 2014 with the objective of safely reducing PTB. The WA Initiative is uniquely the world’s first whole-of-population whole-of-state program aimed at PTB prevention, covering tertiary (one established and one evolving centre that opened during the implementation period), primary and secondary hospitals. In the first year of the Initiative (2015), PTB was reduced by 7.6% across the state and by 20% at the state’s established tertiary centre [7]. A recent study revealed that the PTB reduction up to 2017 was sustained at the state’s established tertiary centre, but not elsewhere in WA [8].

The purpose of this study was to evaluate the effects of the Initiative over the first 5 years (2015–2019), and for the first time, specifically assess outcomes amongst Aboriginal women in WA. It is essential that the Initiative is evaluated amongst Aboriginal women, to gain a better understanding of the major drivers of PTB in this population and thus enable the design of more appropriate and culturally responsive programs.

## Methods

Based on existing evidence, the WA Initiative incorporated new clinical guidelines for singleton pregnancies. This included recommending: measurement of cervix length at all mid-pregnancy morphology scans and administration of vaginal progesterone and/or cervical cerclage for women with a shortened cervix, administration of vaginal progesterone for women with a history of spontaneous PTB, that no pregnancy should be ended prior to 38+ weeks without medical or obstetric justification, identification and provision of smoking cessation counselling to women who smoke, and a new PTB Prevention Clinic at the tertiary centre for high risk cases [7].

We conducted a prospective population-based cohort study using pregnancy data on all Western Australian births from 20 weeks gestation onwards between 2009 and 2019 obtained from the Midwives’ Notification System (MNS) in which pregnancy and birth information is recorded by the attending midwife. Information included data on maternal characteristics (maternal age, health region of residence, socioeconomic status, smoking during pregnancy and ethnicity), medical history (pre-existing diabetes and hypertension, asthma and other pre-existing conditions), obstetric history (parity, previous stillbirth and IVF conception, caesarean at last birth, number of antenatal visits), complications during pregnancy (placental abruption, antepartum haemorrhage, gestational diabetes, threatened miscarriage, threatened preterm labour) and labour and birth (delivery hospital, onset of labour, mode of delivery, gestational age at birth and admission to special care nursery). Socioeconomic status was calculated using the Index of Relative Socio-Economic Advantage and Disadvantage score derived by the Australian Bureau of Statistics [9], and was categorised into those in the lowest 40%, and those in the upper 60%. History of preterm birth was derived for each mother and delivery hospital was used to derive hospital type (established tertiary, evolving tertiary, non-tertiary). Aboriginal status was reported for each individual pregnancy, and mothers who reported being Aboriginal for the majority of their pregnancies were deemed to be Aboriginal for all of them. Given the WA Initiative targeted singleton pregnancies, only singleton pregnancies were included in the analysis. Inductions at the established tertiary hospital before 25 weeks gestation that resulted in intrapartum death were excluded on the assumption that they were terminations of pregnancy.

The PTB rate was examined overall and by gestational age (20–31 weeks and 32–36 weeks). Gestational ages 20–27 and 28–31 were combined due to small number of annual births amongst Aboriginal women. Analyses were performed for the state, for the established tertiary centre, and for non-tertiary hospitals (secondary and primary hospitals combined). Births from the evolving tertiary centre were only included in the state-wide PTB rates as the PTB rate at this centre was substantially different from the established tertiary centre (hereafter referred to as the tertiary centre). Subgroup analyses were also performed by onset of labour (spontaneous/medically initiated) and risk of PTB at the first antenatal visit (low/high).

Binary logistic regression was used to model the likelihood of PTB and stillbirth in each year from 2009 to 2018, relative to these outcomes in 2019, with alternative comparisons using year 2013, the last full year before the Initiative, as the reference. Analyses were performed on pregnancies in years 2009–2019 but only rates from 2013 to 2019 are presented. Supplementary evaluation of PTB incidence was conducted after classifying women into low or high risk of PTB based on information available at the first antenatal visit, using separate logistic regression models for nulliparous and parous women. This risk classification was based on all pregnancies and ethnicities combined, from 2009 to 2019 without year of birth as a predictor, and classification was therefore independent of year of birth. The variables used for classification of low and high risk are shown in Supplementary Table 1.

Patterns of bi-monthly PTB incidence rates were investigated using run charts, which are used to assess temporal changes. Probability rules are used to detect shifts, defined as six consecutive points above or below the baseline rate, and this method is useful where small sample sizes create deficits in power [10], such as among Aboriginal women. For these analyses, the baseline PTB rate was calculated as the median bi-monthly rate from January 2013 to June 2014, which was the 18 months immediately prior to the introduction of the WA Initiative. Run charts by Aboriginal status and hospital type were created by onset of labour and by risk category, for each gestational age group (20–31 weeks, 32–36 weeks, < 37 weeks). There were insufficient numbers of births among Aboriginal women to produce run charts by risk or onset of labour for the 20–31 week gestational age group. This was also the case for low risk Aboriginal women giving birth at the tertiary centre in all gestational age groups. The proportion of high risk women, as well as changes in the rates of risk factors over time were examined by ethnicity and hospital.

SAS statistical software (version 9.4, Cary, NC: SAS Institute Inc.) was used for data analysis with *p*-values< 0.05 considered statistically significant.

The study was approved by the Women and Newborn Health Service Human Research Ethics Committee (RGS0000002677), the Health Department of Western Australia (RGS0000000704) and the Western Australian Aboriginal Health Ethics Committee (965).

## Results

### Pregnancy characteristics in Aboriginal and non-Aboriginal mothers

Table 1 shows the number of births across the study period in the state’s established tertiary centre, evolving tertiary centre and all other hospitals (non-tertiary) by Aboriginal and non-Aboriginal ethnicity, and overall. Overall, the number of singleton births in Western Australia increased from 30,245 in 2009 to 32,272 in 2019. Births to Aboriginal women increased from 1769 in 2009 to 1801 in 2019, while births to non-Aboriginal women increased from 28,476 in 2009 to 30,471 in 2019. Due to the opening of the state’s evolving tertiary centre, the proportion of Aboriginal births that occurred at the state’s established tertiary centre decreased from 27.5% in 2009 to 20.9% in 2019.

**Table 1:** Number of singleton births and PTB in Western Australia, by ethnicity, year and hospital type (2009–2019)

	Births			Established tertiary centre			Evolving tertiary centre			Non-tertiary centres		
	*N*	PTB (*n*)	PTB (%)	*N*	PTB (*n*)	PTB (%)	*N*	PTB (*n*)	PTB (%)	*N*	PTB (*n*)	PTB (%)
**State**												
2009	30,245	2068	6.8	5426	1049	19.3	–	–	–	24,819	1019	4.1
2010	30,363	2127	7.0	5517	987	17.9	–	–	–	24,846	1140	4.6
2011	31,243	2156	6.9	5422	991	18.3	–	–	–	25,821	1165	4.5
2012	32,889	2400	7.3	5681	1086	19.1	–	–	–	27,208	1314	4.8
2013	33,410	2466	7.4	5469	1138	20.8	–	–	–	27,941	1328	4.8
2014	34,129	2405	7.0	5490	1068	19.5	114	2	1.8	28,525	1335	4.7
2015	33,949	2323	6.8	5328	873	16.4	2196	205	9.3	26,425	1245	4.7
2016	34,871	2508	7.2	5321	950	17.9	2727	224	8.2	26,823	1334	5.0
2017	33,450	2547	7.6	5468	983	18.0	2995	247	8.2	24,987	1317	5.3
2018	32,486	2447	7.5	5271	947	18.0	3180	260	8.2	24,035	1240	5.2
2019	32,272	2383	7.4	5405	875	16.2	3045	283	9.3	23,822	1225	5.1
**Non-Aboriginal or Torres Strait Islander Women**												
2009	28,476	1831	6.4	4939	910	18.4	–	–	–	23,537	921	3.9
2010	28,649	1892	6.6	5044	848	16.8	–	–	–	23,605	1044	4.4
2011	29,483	1924	6.5	4963	848	17.1	–	–	–	24,520	1076	4.4
2012	31,217	2131	6.8	5258	945	18	–	–	–	25,959	1186	4.6
2013	31,639	2214	7	5059	1011	20	–	–	–	26,580	1203	4.5
2014	32,337	2166	6.7	5098	949	18.6	108	1	0.9	27,131	1216	4.5
2015	32,213	2092	6.5	4967	761	15.3	2114	189	8.9	25,132	1142	4.5
2016	33,048	2258	6.8	4941	828	16.8	2627	208	7.9	25,480	1222	4.8
2017	31,662	2289	7.2	5103	861	16.9	2891	222	7.7	23,668	1206	5.1
2018	30,706	2189	7.1	4870	825	16.9	3062	241	7.9	22,774	1123	4.9
2019	30,471	2105	6.9	5028	742	14.8	2935	262	8.9	22,508	1101	4.9
**Aboriginal and Torres Strait Islander Women**												
2009	1769	237	13.4	487	139	28.5	–	–	–	1282	98	7.6
2010	1714	235	13.7	473	139	29.4	–	–	–	1241	96	7.7
2011	1760	232	13.2	459	143	31.2	–	–	–	1301	89	6.8
2012	1672	269	16.1	423	141	33.3	–	–	–	1249	128	10.2
2013	1771	252	14.2	410	127	31	–	–	–	1361	125	9.2
2014	1792	239	13.3	392	119	30.4	6	1	16.7	1394	119	8.5
2015	1736	231	13.3	361	112	31	82	16	19.5	1293	103	8
2016	1823	250	13.7	380	122	32.1	100	16	16	1343	112	8.3
2017	1788	258	14.4	365	122	33.4	104	25	24	1319	111	8.4
2018	1780	258	14.5	401	122	30.4	118	19	16.1	1261	117	9.3
2019	1801	278	15.4	377	133	35.3	110	21	19.1	1314	124	9.4

470 terminations performed between 20 and 24 pregnancy weeks at the established tertiary centre were excluded (47, 52, 45, 37, 44, 45, 46, 42, 42, 42, 28 in the respective years from 2009 to 2019)

A summary of maternal characteristics of Aboriginal and non-Aboriginal women are presented in Table 2. Aboriginal women were younger, had more children, were more likely to smoke during pregnancy, reside in in rural areas, and fall in the two lowest socio-economic quintiles. Aboriginal women were also more likely to have pre-existing diabetes, hypertension and other pre-existing conditions, and to have a history of prior PTB or stillbirth. They were less likely to have conceived using IVF or to have had a caesarean at their last delivery. Aboriginal women were more likely to have spontaneous labour than non-Aboriginal women, and their babies were more likely to be admitted to the special care nursery.

**Table 2:** State-wide obstetric risk profile and rates of preterm birth in singleton pregnancies, by ethnicity (2009–2019)

		Non-Aboriginal or Torres Strait Islander women (*N* = 339,901)				Aboriginal and Torres Strait Islander women (*N* = 19,406)				*p*
		*N*	Column	PTB	PTB	*N*	Column	PTB	PTB	
			(%)	(*n*)	(%)		(%)	(*n*)	(%)	
**Maternal Demographics**										
Maternal Age	20–34	256,424	75.4	16,508	6.4	14,256	73.5	1957	13.7	<.001
	< 20	8303	2.4	752	9.1	3512	18.1	471	13.4	
	≥35	75,174	22.1	5831	7.8	1638	8.4	311	19	
Parity	1–4	189,441	55.7	11,970	6.3	11,944	61.5	1720	14.4	<.001
	0	146,525	43.1	10,679	7.3	5893	30.4	741	12.6	
	≥5	3935	1.2	442	11.2	1569	8.1	278	17.7	
Smoked During Pregnancy	No	311,586	91.7	20,110	6.5	10,438	53.8	1243	11.9	<.001
	Yes	28,315	8.3	2981	10.5	8968	46.2	1496	16.7	
Socio-Economic Indexes for Areas (SEIFA) in Lowest 40%	No	279,904	82.3	18,836	6.7	9646	49.7	1396	14.5	<.001
	Yes	57,305	16.9	4070	7.1	9692	49.9	1326	13.7	
	Unknown	2692	0.8	185	6.9	68	0.4	17	25	
**Maternal Conditions**										
Pre-Existing Diabetes	No	337,637	99.3	22,428	6.6	18,929	97.5	2547	13.5	<.001
	Yes	2264	0.7	663	29.3	477	2.5	192	40.3	
Pre-Existing Hypertension	No	336,308	98.9	22,446	6.7	19,156	98.7	2647	13.8	0.002
	Yes	3593	1.1	645	18	250	1.3	92	36.8	
Asthma	No	309,597	91.1	20,754	6.7	17,626	90.8	2499	14.2	0.222
	Yes	30,304	8.9	2337	7.7	1780	9.2	240	13.5	
Other Pre-Existing Conditions	No	224,030	65.9	13,825	6.2	11,377	58.6	1529	13.4	<.001
	Yes	115,871	34.1	9266	8	8029	41.4	1210	15.1	
**Obstetric History**										
Previous Preterm Birth	No	228,907	67.3	14,251	6.2	9680	49.9	1103	11.4	<.001
	Yes	9951	2.9	2373	23.8	1519	7.8	530	34.9	
	Unknown	101,043	29.7	6467	6.4	8207	42.3	1106	13.5	
Previous Stillbirth	0	335,990	98.8	22,424	6.7	18,857	97.2	2578	13.7	<.001
	1	3640	1.1	613	16.8	505	2.6	143	28.3	
	≥2	271	0.1	54	19.9	44	0.2	18	40.9	
Caesarean Last Delivery	No	282,473	83.1	18,568	6.6	16,686	86	2306	13.8	<.001
	Yes	57,428	16.9	4523	7.9	2720	14	433	15.9	
In vitro fertilization Conception	No	328,693	96.7	21,933	6.7	19,349	99.7	2736	14.1	<.001
	Yes	11,208	3.3	1158	10.3	57	0.3	< 5	< 5.5	
**Delivery Hospital**										
Hospital	Non-tertiary centres	270,894	79.7	12,440	4.6	14,358	74	1222	8.5	<.001
	Established tertiary	55,270	16.3	9528	17.2	4528	23.3	1419	31.3	
	Evolving tertiary	13,737	4	1123	8.2	520	2.7	98	18.8	
Metropolitan/Rural	Metropolitan	289,532	85.2	21,240	7.3	9512	49	1873	19.7	<.001
	Rural	50,365	14.8	1850	3.7	9881	50.9	859	8.7	
	Unknown	< 5	0	< 5	25.0	13	0.1	7	53.8	
**Labour, Birth and Neonatal**										
Number of Antenatal Visits*	0	172	0.1			96	0.9			<.001
	1–6	23,786	12.5			3722	35.15			
	7–19	165,161	86.5			6644	62.7			
	20+	1798	0.9			127	1.2			
Onset of Labour	Spontaneous	161,440	47.5	10,703	6.6	11,654	60.1	1636	14	<.001
	Induced	104,802	30.8	5368	5.1	5131	26.4	588	11.5	
	No Labour	73,653	21.7	7019	9.5	2620	13.5	514	19.6	
	Unknown	6	0	< 5	16.7	< 5	0	< 5	100	
Admitted to Special Care Nursery	No	304,367	89.5	10,210	3.4	16,642	85.8	1345	8.1	<.001
	Yes	35,534	10.5	12,881	36.2	2764	14.2	1394	50.4	
**Region of Residence**^**+**^										
	All Metro	274,105	80.6	19,037	6.9	7216	37.2	1105	15.3	<.001
	South West and Great Southern	31,371	9.2	1880	5.99	1605	8.3	209	13.02	
	Midwest	7283	2.1	472	6.5	2076	10.7	284	13.7	
	Goldfields	8572	2.5	584	6.8	1496	7.7	217	14.5	
	Wheatbelt	6053	1.8	414	6.8	649	3.3	96	14.8	
	Kimberley	2898	0.9	146	5	4174	21.5	492	11.8	
	Pilbara	7457	2.2	381	5.1	2147	11.1	323	15	
**High risk for PTB at first antenatal visit**										
	Low risk	284,590	83.7	15,742	5.5	3309	17.1	277	8.4	<.001
	High risk	55,311	16.3	7349	13.3	16,097	82.9	2462	15.3	

*Only term births from 2013 are included

^+^Column percentages don’t add up to 100 due to missing data

### Risk factors for PTB, by hospital and year, for Aboriginal and non-Aboriginal mothers

Across the study period, PTB rates were higher amongst Aboriginal women with an overall PTB rate of 14.1% compared to 6.8% amongst non-Aboriginal women. Pregnancies were classified as being at low or high risk of PTB based on information available at the first antenatal visit, and the clinical profile described above resulted in 82.9% of Aboriginal women being classified as high risk, compared to 16.3% of non-Aboriginal women. The PTB rate was 15.3% amongst high risk Aboriginal women compared to 13.3% amongst high risk non-Aboriginal women. Amongst low risk Aboriginal women, the PTB rate was 8.4% compared to 5.5% amongst low risk non-Aboriginal women. The PTB rates for the variables used for classification, by risk level are presented in Supplementary Table 1. Women classified as high risk of PTB also experienced higher rates of pregnancy complications including stillbirth, threatened preterm labour, gestational diabetes, pre-eclampsia, antepartum haemorrhage and preterm pre-labour rupture of membranes with delivery < 37 weeks, irrespective of ethnicity (Supplementary Table 2).

Amongst non-Aboriginal women, the proportion of high risk women increased from 15.9% in 2009 to 16.8% in 2019 which was equivalent to an additional 270 women in 2019 compared to 2009. The proportion of high risk women increased slightly more at the tertiary hospital (22.7% in 2009 vs 25.2% in 2019, an additional 124 women) than at non-tertiary hospitals (14.4% in 2009 vs 14.8% in 2019, an additional 75 women women). The proportion of Aboriginal women classified as high risk decreased from 84.2% in 2009 to 78.6% in 2019, which was equivalent to a reduction of 99 women in 2019 compared to 2009. The proportion of high risk Aboriginal women birthing at the tertiary hospital was higher than at non-tertiary hospitals, with a similar percentage decrease across the two hospital types (91.6%% in 2009 vs 85.1% in 2019, a reduction of 24 women in the tertiary hospital, compared to 81.4% in 2009 vs 76.9% in 2019, a reduction of 59 women in non-tertiary hospitals). The major risk factor that decreased in this Aboriginal population was smoking during pregnancy, decreasing across the state from 51.1% of women in 2009, to 42.4% of women in 2019, with almost all of the decrease occurring in non-tertiary hospitals. Concurrently, the proportion of Aboriginal women who attended their first antenatal visit in the first trimester of pregnancy increased from 46.8% in 2013 to 51.0% in 2019.

### PTB rates in Aboriginal and non-Aboriginal mothers

Table 3 shows the annual rates of PTB among Aboriginal and non-Aboriginal women in the tertiary centre, non-tertiary hospitals combined and state-wide from 2013 to 2019.

**Table 3:** Rates of preterm birth in singleton pregnancies stratified by gestational age, ethnicity and hospital, 2013–2019

Gestational age/year	Non-aboriginal or Torres Strait Islander women									Aboriginal and Torres Strait Islander women								
	Tertiary centre			Non-tertiary centres			State			Tertiary centre			Non-tertiary centres			State		
	*N*	*n*	%	*N*	*n*	%	*N*	*n*	%	*N*	*n*	%	*N*	*n*	%	*N*	*n*	%
**20–31 Weeks**																		
2013	5059	262	5.2	26,580	63	0.2	31,639	325	1	410	33	8	1361	15	1.1	1771	48	2.7
2014	**5098**	**281**	**5.5**^**↑**^	27,131	57	0.2	32,337	338	1	392	37	9.4	1394	14	1	1792	51	2.8
2015	4967	225	4.5	25,132	50	0.2	32,213	288	0.9	361	31	8.6	1293	12	0.9	1736	45	2.6
2016	**4941**	**270**	**5.5**^**↑**^	25,480	71	0.3	33,048	350	1.1	380	38	10	1343	16	1.2	1823	55	3
2017	5103	236	4.6	23,668	62	0.3	31,662	322	1	365	30	8.2	1319	14	1.1	1788	45	2.5
2018	4870	227	4.7	22,774	55	0.2	30,706	301	1	401	37	9.2	1261	12	1	1780	52	2.9
2019	5028	226	4.5	22,508	61	0.3	30,471	323	1.1	377	41	10.9	1314	15	1.1	1801	59	3.3
**32–36 Weeks**																		
2013	**5059**	**749**	**14.8**^**↑**^	26,580	1140	4.3	31,639	1889	6	410	94	22.9	1361	110	8.1	1771	204	11.5
2014	**5098**	**668**	**13.1**^**↑**^	**27,131**	**1159**	**4.3**^**↓**^	32,337	1828	5.7	392	82	20.9	1394	105	7.5	1792	188	10.5
2015	4967	536	10.8	25,132	1092	4.3	32,213	1804	5.6	361	81	22.4	1293	91	7	1736	186	10.7
2016	4941	558	11.3	25,480	1151	4.5	33,048	1908	5.8	380	84	22.1	1343	96	7.1	1823	195	10.7
2017	**5103**	**625**	**12.2**^**↑**^	23,668	1144	4.8	**31,662**	**1967**	**6.2**^**↑**^	365	92	25.2	1319	97	7.4	1788	213	11.9
2018	**4870**	**598**	**12.3**^**↑**^	22,774	1068	4.7	**30,706**	**1888**	**6.1**^**↑**^	401	85	21.2	1261	105	8.3	1780	206	11.6
2019	5028	516	10.3	22,508	1040	4.6	30,471	1782	5.8	377	92	24.4	1314	109	8.3	1801	219	12.2
**< 37 Weeks**																		
2013	**5059**	**1011**	**20.0**^**↑**^	**26,580**	**1203**	**4.5**^**↓**^	31,639	2214	7	410	127	31.0	1361	125	9.2	1771	252	14.2
2014	**5098**	**949**	**18.6**^**↑**^	**27,131**	**1216**	**4.5**^**↓**^	32,337	2166	6.7	392	119	30.4	1394	119	8.5	1792	239	13.3
2015	4967	761	15.3	25,132	1142	4.5	32,213	2092	6.5	361	112	31.0	1293	103	8	**1736**	**231**	**13.3**^**↓**^
2016	**4941**	**828**	**16.8**^**↑**^	25,480	1222	4.8	33,048	2258	6.8	380	122	32.1	1343	112	8.3	1823	250	13.7
2017	**5103**	**861**	**16.9**^**↑**^	23,668	1206	5.1	31,662	2289	7.2	365	122	33.4	1319	111	8.4	1788	258	14.4
2018	**4870**	**825**	**16.9**^**↑**^	22,774	1123	4.9	30,706	2189	7.1	401	122	30.4	1261	117	9.3	1780	258	14.5
2019	5028	742	14.8	22,508	1101	4.9	30,471	2105	6.9	377	133	35.3	1314	124	9.4	1801	278	15.4

-PTB rates are compared using logistic regression analysis with year 2019 as a reference. Statistical adjustments were made for all maternal characteristics known at the time of the first antenatal visit used to derive low risk of PTB including maternal age, pre-existing diabetes, pre-existing hypertension, other pre-existing maternal medical conditions, asthma, smoking during pregnancy, ethnicity (Aboriginal, Caucasian, other), socioeconomic status in the lowest two quintiles, IVF conception, history of stillbirth, history of PTB and caesarean at last delivery. Rates that were significantly different from 2019 after adjustments for maternal characteristics are shown by ↓ for lower rates and ↑ for higher rates relative to the 2019 rates

**Fig. 1:**
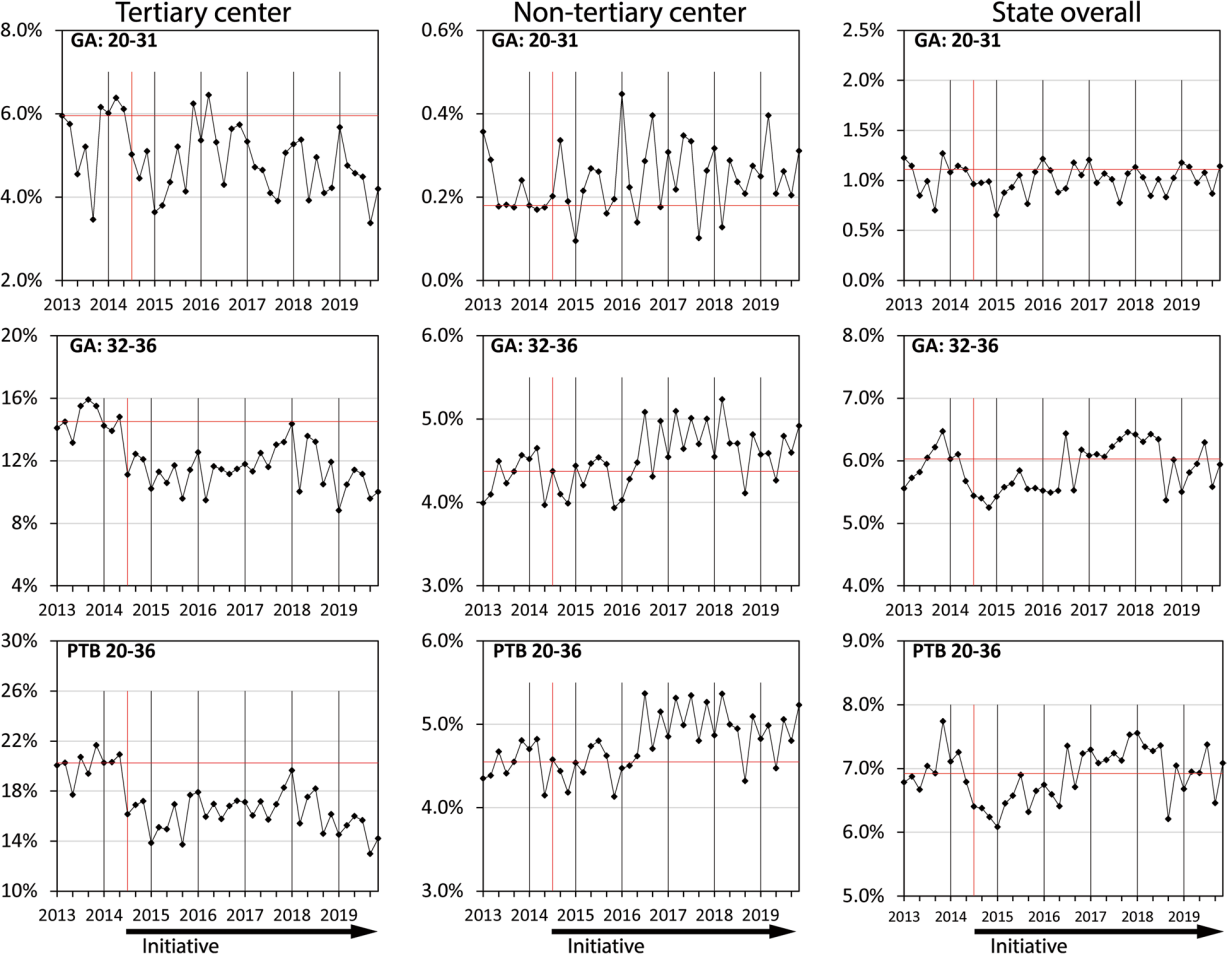
Rates of preterm birth in singleton pregnancies, by gestational age and hospital level, non-Aboriginal Women The minimum and maximum number of births per two month time epoch were: Tertiary centre: 743, 903, Non-tertiary centre: 3537, 4755, State overall: 4813, 5717

**Fig. 2:**
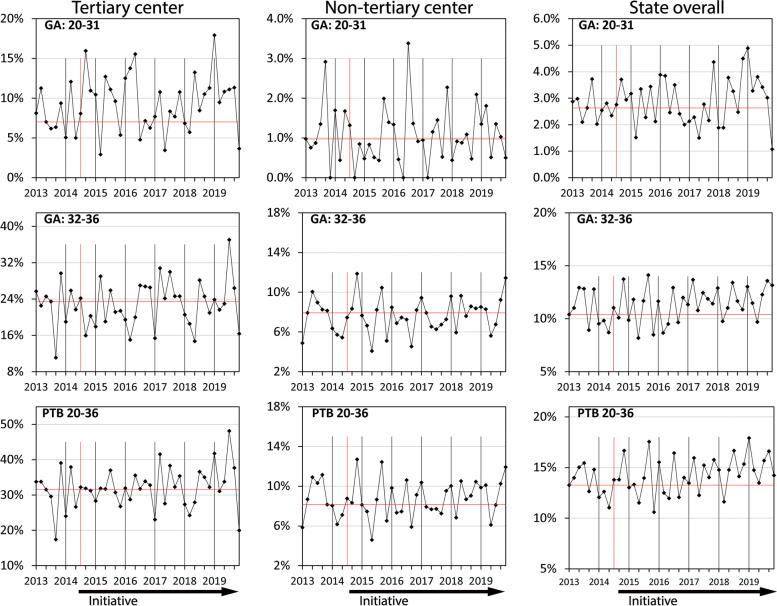
Rates of preterm birth in singleton pregnancies, by gestational age and hospital level, Aboriginal Women The minimum and maximum number of births per two month time epoch were: Tertiary centre: 45, 81, Non-tertiary centre: 184, 277, State overall: 262, 312

For non-Aboriginal women at the state’s tertiary centre, the rate of PTB was significantly reduced from 20.0% in 2013 to its lowest rate of 14.8% in 2019 (*p* < 0.001). The significant reduction in the PTB rate occurred primarily in births between 32 and 36 weeks which decreased from 14.8% in 2013 to a minimum of 10.3% in 2019 (*p* < 0.001). Run charts of PTB incidence amongst non-Aboriginal women by gestational age group demonstrated a significant reduction in PTB for all years of the Initiative (Fig. 1). No similar improvements occurred amongst Aboriginal women for whom the PTB rate increased from 31.0% in 2013 to 35.3% in 2019 (*p* = 0.393) and run chart analysis showed an increase in PTB between 20 and 31 weeks during 2018 and 2019 (Fig. 2).

For non-Aboriginal women in non-tertiary hospitals, the PTB rate increased from 4.5% in 2013 to 5.1% in 2017 (*p* = 0.001), to 4.9% in 2018 (*p* = 0.004) and 2019 (*p* = 0.032). The increase was primarily driven by births between 32 and 36 weeks where PTB increased from 4.3% in 2013 to 4.8% in 2017 (*p* = 0.001) and 4.7% in 2018 (*p* = 0.004). Run charts showed an increase in PTB among births between 20 and 31 weeks in 2018 and 2019, and in all PTB and PTB between 32 and 36 weeks from 2016 to 2018 (Fig. 1). Amongst Aboriginal women in non-tertiary hospitals, the PTB rate was 9.2% in 2013 and in subsequent years ranged from a minimum of 8.0% in 2015 (*p* = 0.222) to a maximum of 9.4% in 2019 (*p* = 0.867). Run chart analysis showed an increase in overall PTB during 2018 and at the start of 2019 (Fig. 2).

For non-Aboriginal women across the state the PTB rate was 7.0% in 2013, and subsequently ranged from 6.5% in 2015 (*p* = 0.017) to 7.2% in 2017 (*p* = 0.372). In 2018 and 2019 the PTB rates were similar to pre-Initiative levels at 7.1% (*p* = 0.370) and 6.9% (*p* = 0.404) respectively. Run charts showed a reduction in PTB in all gestational age groups for the two years after the introduction of the Initiative. Subsequently, increases in PTB incidence were observed between 2016 and 2018 for the 32–36 week gestational age group, and overall (Fig. 1). Amongst Aboriginal women across the state, the PTB rate was 14.2% in 2013, with the lowest rate occurring in 2015, the first full year of the Initiative (13.3%, *p* = 0.236) and the highest in 2019, the most recent year of data (15.4%, *p* = 0.337). Run charts showed increases in PTB incidence in all gestational age groups during 2018 and 2019, and also during 2016 and 2017 in births between 32 and 36 weeks (Fig. 2).

### PTB rates in Aboriginal and non-Aboriginal mothers by risk

Tables 4 and 5 show the annual rates of PTB among Aboriginal and non-Aboriginal women in the tertiary centre, non-tertiary hospitals combined and state-wide from 2013 to 2019, by low and high risk of PTB.

**Table 4:** Rates of preterm birth in low risk singleton pregnancies, by gestational age, ethnicity and hospital level

Gestational age/year	Non-aboriginal or Torres Strait Islander women									Aboriginal and Torres Strait Islander women								
	Tertiary centre			Non-tertiary centres			State			Tertiary centre			Non-tertiary centres			State		
	*N*	*n*	%	*N*	*n*	%	*N*	*n*	%	*N*	*n*	%	*N*	*n*	%	*N*	*n*	%
**20–31 Weeks**																		
2013	3785	166	4.4	22,679	45	0.2	26,464	211	0.8	23	< 5	< 21.7	234	< 5	< 2.1	257	< 5	< 1.9
2014	**3785**	**186**	**4.9**^**↑**^	23,079	46	0.2	26,960	232	0.9	32	< 5	< 15.6	240	< 5	< 2.1	274	< 5	< 1.8
2015	3705	150	4.0	21,550	40	0.2	27,090	198	0.7	39	< 5	< 12.8	235	< 5	< 2.1	288	7	2.4
2016	**3649**	**169**	**4.6**^**↑**^	21,730	54	0.2	27,620	228	0.8	28	< 5	< 17.9	251	< 5	< 2.0	300	< 5	< 1.7
2017	3768	135	3.6	20,184	51	0.3	26,376	202	0.8	28	< 5	< 17.9	274	< 5	< 1.8	324	5	1.5
2018	3666	147	4.0	19,700	48	0.2	25,918	210	0.8	50	< 5	< 10.0	289	< 5	< 1.7	358	6	1.7
2019	3763	138	3.7	19,186	45	0.2	25,366	204	0.8	56	7	12.5	304	< 5	< 1.6	385	9	2.3
**32–36 Weeks**																		
2013	**3785**	**467**	**12.3**^**↑**^	22,679	841	3.7	26,464	1308	4.9	23	< 5	< 21.7	234	15	6.4	257	17	6.6
2014	**3785**	**392**	**10.4**^**↑**^	23,079	829	3.6	26,960	1222	4.5	32	5	15.6	240	15	6.3	274	20	7.3
2015	3705	296	8.0	21,550	798	3.7	27,090	1223	4.5	39	7	17.9	235	14	6.0	288	23	8.0
2016	3649	322	8.8	21,730	808	3.7	27,620	1277	4.6	28	5	17.9	251	10	4.0	300	17	5.7
2017	**3768**	**359**	**9.5**^**↑**^	20,184	793	3.9	26,376	1281	4.9	28	< 5	< 17.9	274	12	4.4	324	17	5.2
2018	**3666**	**345**	**9.4**^**↑**^	19,700	770	3.9	25,918	1271	4.9	50	7	14.0	289	14	4.8	358	22	6.1
2019	3763	302	8.0	19,186	709	3.7	25,366	1159	4.6	56	8	14.3	304	21	6.9	385	33	8.6
**< 37 Weeks**																		
2013	**3785**	**633**	**16.7**^**↑**^	22,679	886	3.9	26,464	1519	5.7	23	< 5	< 21.7	234	17	7.3	**257**	**19**	**7.4**^**↓**^
2014	**3785**	**578**	**15.3**^**↑**^	23,079	875	3.8	26,960	1454	5.4	32	8	25.0	240	15	6.3	274	23	8.4
2015	3705	446	12.0	21,550	838	3.9	27,090	1421	5.2	39	11	28.2	235	17	7.2	288	30	10.4
2016	**3649**	**491**	**13.5**^**↑**^	21,730	862	4.0	27,620	1505	5.4	28	8	28.6	251	10	4.0	**300**	**20**	**6.7**^**↓**^
2017	**3768**	**494**	**13.1**^**↑**^	20,184	844	4.2	26,376	1483	5.6	28	< 5	< 17.9	274	16	5.8	**324**	**22**	**6.8**^**↓**^
2018	**3666**	**492**	**13.4**^**↑**^	19,700	818	4.2	25,918	1481	5.7	50	11	22.0	289	16	5.5	358	28	7.8
2019	3763	440	11.7	19,186	754	3.9	25,366	1363	5.4	56	15	26.8	304	22	7.2	385	42	10.9

-PTB rates are compared using logistic regression analysis with year 2019 as a reference. Statistical adjustments were made for all maternal characteristics known at the time of the first antenatal visit used to derive low risk of PTB including maternal age, pre-existing diabetes, pre-existing hypertension, other pre-existing maternal medical conditions, asthma, smoking during pregnancy, ethnicity (Aboriginal, Caucasian, other), socioeconomic status in the lowest two quintiles, IVF conception, history of stillbirth, history of PTB and caesarean at last delivery. Rates that were significantly different from 2019 after adjustments for maternal characteristics are shown by ↓ for lower rates and ↑ for higher rates relative to the 2019 rates. PTB cases were supressed when < 5 cases occurred and the upper bounds for these rates were calculated based on 5 cases

**Table 5:** Rates of preterm birth in high risk singleton pregnancies, by gestational age, ethnicity and hospital level

Gestational age/year	Non-aboriginal or Torres Strait Islander women									Aboriginal and Torres Strait Islander women								
	Tertiary centre			Non-tertiary centres			State			Tertiary centre			Non-tertiary centres			State		
	*N*	*n*	%	*N*	*n*	%	*N*	*n*	%	*N*	*n*	%	*N*	*n*	%	*N*	*n*	%
**20–31 Weeks**																		
2013	1274	96	7.5	3901	18	0.5	5175	114	2.2	387	33	8.5	1127	13	1.2	1514	46	3.0
2014	1313	95	7.2	4052	11	0.3	5377	106	2.0	360	34	9.4	1154	14	1.2	1518	48	3.2
2015	1262	75	5.9	3582	10	0.3	5123	90	1.8	322	27	8.4	1058	9	0.9	1448	38	2.6
2016	1292	101	7.8	3750	17	0.5	5428	122	2.2	352	35	9.9	1092	16	1.5	1523	52	3.4
2017	1335	101	7.6	3484	11	0.3	5286	120	2.3	337	29	8.6	1045	10	1.0	1464	40	2.7
2018	1204	80	6.6	3074	7	0.2	4788	91	1.9	351	33	9.4	972	10	1.0	1422	46	3.2
2019	1265	88	7.0	3322	16	0.5	5105	119	2.3	321	34	10.6	1010	14	1.4	1416	50	3.5
**32–36 Weeks**																		
2013	**1274**	**282**	**22.1**^**↑**^	3901	299	7.7	5175	581	11.2	387	92	23.8	1127	95	8.4	1514	187	12.4
2014	**1313**	**276**	**21.0**^**↑**^	4052	330	8.1	5377	606	11.3	360	77	21.4	1154	90	7.8	1518	168	11.1
2015	1262	240	19.0	3582	294	8.2	5123	581	11.3	322	74	23.0	1058	77	7.3	1448	163	11.3
2016	1292	236	18.3	3750	343	9.1	5428	631	11.6	352	79	22.4	1092	86	7.9	1523	178	11.7
2017	1335	266	19.9	3484	351	10.1	5286	686	13.0	337	89	26.4	1045	85	8.1	1464	196	13.4
2018	1204	253	21.0^↑^	3074	298	9.7	4788	617	12.9	351	78	22.2	972	91	9.4	1422	184	12.9
2019	1265	214	16.9	3322	331	10.0	5105	623	12.2	321	84	26.2	1010	88	8.7	1416	186	13.1
**< 37 Weeks**																		
2013	**1274**	**378**	**29.7**^**↑**^	3901	317	8.1	5175	695	13.4	387	125	32.3	1127	108	9.6	1514	233	15.4
2014	**1313**	**371**	**28.3**^**↑**^	4052	341	8.4	5377	712	13.2	360	111	30.8	1154	104	9.0	1518	216	14.2
2015	1262	315	25.0	**3582**	**304**	**8.5**^**↓**^	5123	671	13.1	322	101	31.4	1058	86	8.1	**1448**	**201**	**13.9**^**↓**^
2016	1292	337	26.1	3750	360	9.6	5428	753	13.9	352	114	32.4	1092	102	9.3	1523	230	15.1
2017	1335	367	27.5	3484	362	10.4	5286	806	15.2	337	118	35.0	1045	95	9.1	1464	236	16.1
2018	1204	333	27.7	3074	305	9.9	4788	708	14.8	351	111	31.6	972	101	10.4	1422	230	16.2
2019	1265	302	23.9	3322	347	10.4	5105	742	14.5	321	118	36.8	1010	102	10.1	1416	236	16.7

-PTB rates are compared using logistic regression analysis with year 2019 as a reference. Statistical adjustments were made for all maternal characteristics known at the time of the first antenatal visit used to derive low risk of PTB including maternal age, pre-existing diabetes, pre-existing hypertension, other pre-existing maternal medical conditions, asthma, smoking during pregnancy, ethnicity (Aboriginal, Caucasian, other), socioeconomic status in the lowest two quintiles, IVF conception, history of stillbirth, history of PTB and caesarean at last delivery. Rates that were significantly different from 2019 after adjustments for maternal characteristics are shown by ↓ for lower rates and ↑ for higher rates relative to the 2019 rates

For non-Aboriginal women at the state’s tertiary centre, who were classified at low risk, the PTB rate declined from 16.7% in 2013 in every subsequent year, with the reduction reaching statistical significance in years 2015–2019. The lowest PTB rate occurred in 2019 (11.7%, *p* < 0.001) and the reduction over time was primarily due to a reduction in PTB between 32 and 36 weeks. Run charts of PTB incidence, by gestational age group demonstrated a significant reduction in PTB overall and from 32 to 36 weeks for all years of the Initiative, and from 2016 to 2019 for PTB between 20 and 31 weeks (Supplementary Fig. 1). For high risk non-Aboriginal women who gave birth at the state’s tertiary centre, the PTB rate decreased from 29.7% in 2013, in every subsequent year, with the reduction reaching statistical significance from 2015 to 2019. The PTB rate of 23.9% was at its minimum in 2019 (*p* < 0.001), although this was a smaller percentage decrease (19.5%) than that in low risk births (30.1%). Run charts showed a reduction in PTB incidence overall and from 32 to 36 weeks for all years of the initiative (Supplementary Fig. 1).

For non-Aboriginal women at non-tertiary hospitals who were classified at low risk, the PTB rate increased from 3.9% in 2013, to 4.2% in 2017 (*p* = 0.011) and 2018 (*p* = 0.013). This was due to an increase in PTB from 32 to 36 weeks from 3.7% in 2013 to 3.9% in 2017 (*p* = 0.027) and 2018 (*p* = 0.029). Run charts by gestational age group demonstrated a significant increase in PTB incidence from 20 to 31 weeks in 2018 and 2019 (Supplementary Fig. 1). For non-Aboriginal women at non-tertiary hospitals who were at high risk, the PTB rate was higher than in 2013 (8.1%) in every subsequent year, with this difference reaching statistical significance in 2017 only (10.4%, *p* = 0.031). Between 2013 to 2019 there was a larger percentage increase amongst women at high risk of PTB (28.5%) compared to women at low risk of PTB (0.6%). Run charts by gestational age group demonstrated a significant increase in PTB incidence overall and from 32 to 36 weeks between 2016 and 2019 (Supplementary Fig. 1).

For Aboriginal women at the tertiary centre who were classified at low risk, evaluation of the PTB rate is limited by small numbers ranging from just 23 births in 2013 to 56 births in 2019. This is reflected in the unstable PTB rate that was 8.7% in 2013 rising to a maximum of 28.6% (*p* = 0.116) in 2016 (Table 4). These low numbers also prevented meaningful run chart analysis. For Aboriginal women at the tertiary centre who were classified at high risk, the PTB rate was 32.3% in 2013 and then ranged between 30.8% in 2014 (*p* = 0.585) and 36.8% in 2019 (*p* = 0.611). Run charts by gestational age group amongst high risk Aboriginal women at the tertiary centre demonstrated a reduction in the overall PTB rate from 2014 to 2015 (Supplementary Fig. 2).

For low risk Aboriginal women at non-tertiary hospitals, the PTB rate was 7.3% in 2013, then ranged between a minimum of 4.0% in 2016 (*p* = 0.222) and a maximum of 7.2% in 2015 (*p* = 0.792) and 2019 (*p* = 0.516). For Aboriginal women at non-tertiary hospitals who were classified at high risk, following a rate of 9.6% in 2013, the PTB rate was at its lowest at 8.1% in 2015 (*p* = 0.154) and highest at 10.4% in 2018 (*p* = 0.884). Run charts by gestational age group amongst high risk Aboriginal women at non-tertiary hospitals demonstrated an increase in PTB overall and among births between 32 and 36 weeks in years 2018 and 2019 (Supplementary Fig. 2).

### PTB rates in Aboriginal and non-Aboriginal women by onset of labour

Tables 6 and 7 show the annual rates of PTB among Aboriginal and non-Aboriginal women, by onset of labour, in the tertiary centre, non-tertiary hospitals combined and state-wide from 2013 to 2019.

**Table 6:** Rates of medically initiated preterm birth in singleton pregnancies, by gestational age, ethnicity and hospital level

Gestational age /year	Non-aboriginal or Torres Strait Islander women									Aboriginal and Torres Strait Islander women								
	Tertiary centre			Non-tertiary centres			State			Tertiary centre			Non-tertiary centres			State		
	*N*	*n*	%	*N*	*n*	%	*N*	*n*	%	*N*	*n*	%	*N*	*n*	%	*N*	*n*	%
**20–31 Weeks**																		
2013	**5059**	**163**	**3.2**^**↑**^	26,580	31	0.1	31,639	194	0.6	410	17	4.1	1361	< 5	0.1	1771	19	1.1
2014	5098	151	3	27,131	25	0.1	32,337	176	0.5	392	20	5.1	1394	< 5	0.1	1792	21	1.2
2015	4967	125	2.5	25,132	28	0.1	32,213	161	0.5	361	13	3.6	1293	< 5	0	1736	13	0.7
2016	4941	146	3	25,480	39	0.2	33,048	193	0.6	380	14	3.7	1343	< 5	0.1	1823	15	0.8
2017	5103	149	2.9	23,667	37	0.2	31,661	198	0.6	365	19	5.2	1319	< 5	0.3	1788	23	1.3
2018	4870	136	2.8	22,773	28	0.1	30,705	176	0.6	401	16	4	1261	< 5	0.2	1780	22	1.2
2019	5028	135	2.7	22,504	33	0.1	30,467	189	0.6	377	19	5	1313	< 5	0.1	1800	22	1.2
**32–36 Weeks**																		
2013	**5059**	**462**	**9.1**^**↑**^	**26,580**	**507**	**1.9**^**↓**^	**31,639**	**969**	**3.1**^**↓**^	410	57	13.9	**1361**	**25**	**1.8**^**↓**^	1771	82	4.6
2014	5098	401	7.9	**27,131**	**596**	**2.2**^**↓**^	**32,337**	**997**	**3.1**^**↓**^	392	49	12.5	**1394**	**30**	**2.2**^**↓**^	**1792**	**79**	**4.4**^**↓**^
2015	4967	298	6	**25,132**	**556**	**2.2**^**↓**^	**32,213**	**928**	**2.9**^**↓**^	361	33	9.1	**1293**	**25**	**1.9**^**↓**^	**1736**	**63**	**3.6**^**↓**^
2016	4941	355	7.2	25,480	590	2.3	33,048	1037	3.1	380	41	10.8	**1343**	**27**	**2.0**^**↓**^	**1823**	**74**	**4.1**^**↓**^
2017	5103	418	8.2	23,667	612	2.6	31,661	1133	3.6	365	51	14	1319	34	2.6	1788	97	5.4
2018	4870	364	7.5	22,773	607	2.7	30,705	1093	3.6	401	50	12.5	1261	38	3	1780	96	5.4
2019	5028	347	6.9	22,504	570	2.5	30,467	1043	3.4	377	50	13.3	1313	46	3.5	1800	104	5.8
**< 37 Weeks**																		
2013	**5059**	**625**	**12.4**^**↑**^	**26,580**	**538**	**2.0**^**↓**^	**31,639**	**1163**	**3.7**^**↓**^	410	74	18	**1361**	**27**	**2.0**^**↓**^	1771	101	5.7
2014	**5098**	**552**	**10.8**^**↑**^	**27,131**	**621**	**2.3**^**↓**^	**32,337**	**1173**	**3.6**^**↓**^	392	69	17.6	**1394**	**31**	**2.2**^**↓**^	**1792**	**100**	**5.6**^**↓**^
2015	4967	423	8.5	**25,132**	**584**	**2.3**^**↓**^	**32,213**	**1089**	**3.4**^**↓**^	361	46	12.7	**1293**	**25**	**1.9**^**↓**^	**1736**	**76**	**4.4**^**↓**^
2016	4941	501	10.1	25,480	629	2.5	33,048	1230	3.7	380	55	14.5	**1343**	**28**	**2.1**^**↓**^	**1823**	**89**	**4.9**^**↓**^
2017	**5103**	**567**	**11.1**^**↑**^	23,667	649	2.7	31,661	1331	4.2	365	70	19.2	1319	38	2.9	1788	120	6.7
2018	4870	500	10.3	22,773	635	2.8	30,705	1269	4.1	401	66	16.5	1261	41	3.3	1780	118	6.6
2019	5028	482	9.6	22,504	603	2.7	30,467	1232	4	377	69	18.3	1313	47	3.6	1800	126	7

-PTB rates are compared using logistic regression analysis with year 2019 as a reference. Statistical adjustments were made for all maternal characteristics known at the time of the first antenatal visit used to derive low risk of PTB including maternal age, pre-existing diabetes, pre-existing hypertension, other pre-existing maternal medical conditions, asthma, smoking during pregnancy, ethnicity (Aboriginal, Caucasian, other), socioeconomic status in the lowest two quintiles, IVF conception, history of stillbirth, history of PTB and caesarean at last delivery. Rates that were significantly different from 2019 after adjustments for maternal characteristics are shown by ↓ for lower rates and ↑ for higher rates relative to the 2019 rates

**Table 7:** Rates of spontaneous preterm birth in singleton pregnancies, by gestational age, ethnicity and hospital level

Gestational age/year	Non-aboriginal or Torres Strait Islander women									Aboriginal and Torres Strait Islander women								
	Tertiary centre			Non-tertiary centres			State			Tertiary centre			Non-tertiary centres			State		
	*N*	*n*	%	*N*	*n*	%	*N*	*n*	%	*N*	*n*	%	*N*	*n*	%	*N*	*n*	%
**20–31 Weeks**																		
2013	5059	99	2	26,580	32	0.1	31,639	131	0.4	410	16	3.9	1361	13	1	1771	29	1.6
2014	**5098**	**130**	**2.6**^**↑**^	27,131	32	0.1	32,337	162	0.5	392	17	4.3	1394	13	0.9	1792	30	1.7
2015	4967	100	2	25,132	22	0.1	32,213	127	0.4	361	18	5	1293	12	0.9	1736	32	1.8
2016	**4941**	**124**	**2.5**^**↑**^	25,480	32	0.1	33,048	157	0.5	380	24	6.3	1343	15	1.1	1823	40	2.2
2017	5103	87	1.7	23,667	25	0.1	31,661	124	0.4	365	11	3	1319	10	0.8	**1788**	**22**	**1.2**^**↓**^
2018	4870	91	1.9	22,773	27	0.1	30,705	125	0.4	401	21	5.2	1261	9	0.7	1780	30	1.7
2019	5028	91	1.8	22,504	28	0.1	30,467	134	0.4	377	22	5.8	1313	14	1.1	1800	37	2.1
**32–36 Weeks**																		
2013	**5059**	**287**	**5.7**^**↑**^	**26,580**	**633**	**2.4**^**↑**^	**31,639**	**920**	**2.9**^**↑**^	410	37	9	1361	85	6.2	1771	122	6.9
2014	**5098**	**267**	**5.2**^**↑**^	27,131	563	2.1	32,337	831	2.6	392	33	8.4	1394	75	5.4	1792	109	6.1
2015	**4967**	**238**	**4.8**^**↑**^	25,132	536	2.1	**32,213**	**876**	**2.7**^**↑**^	361	48	13.3	1293	66	5.1	1736	123	7.1
2016	4941	203	4.1	25,480	561	2.2	33,048	871	2.6	380	43	11.3	1343	69	5.1	1823	121	6.6
2017	5103	207	4.1	23,667	532	2.2	31,661	834	2.6	365	41	11.2	1319	63	4.8	1788	116	6.5
2018	**4870**	**234**	**4.8**^**↑**^	22,773	461	2	30,705	795	2.6	401	35	8.7	1261	67	5.3	1780	110	6.2
2019	5028	169	3.4	22,504	469	2.1	30,467	738	2.4	377	42	11.1	1313	62	4.7	1800	114	6.3
**< 37 Weeks**																		
2013	**5059**	**386**	**7.6**^**↑**^	**26,580**	**665**	**2.5**^**↑**^	**31,639**	**1051**	**3.3**^**↑**^	410	53	12.9	1361	98	7.2	1771	151	8.5
2014	**5098**	**397**	**7.8**^**↑**^	27,131	595	2.2	32,337	993	3.1	392	50	12.8	1394	88	6.3	1792	139	7.8
2015	**4967**	**338**	**6.8**^**↑**^	25,132	558	2.2	**32,213**	**1003**	**3.1**^**↑**^	361	66	18.3	1293	78	6	1736	155	8.9
2016	**4941**	**327**	**6.6**^**↑**^	25,480	593	2.3	33,048	1028	3.1	380	67	17.6	1343	84	6.3	1823	161	8.8
2017	5103	294	5.8	23,667	557	2.4	31,661	958	3	365	52	14.2	1319	73	5.5	1788	138	7.7
2018	**4870**	**325**	**6.7**^**↑**^	22,773	488	2.1	30,705	920	3	401	56	14	1261	76	6	1780	140	7.9
2019	5028	260	5.2	22,504	497	2.2	30,467	872	2.9	377	64	17	1313	76	5.8	1800	151	8.4

-PTB rates are compared using logistic regression analysis with year 2019 as a reference. Statistical adjustments were made for all maternal characteristics known at the time of the first antenatal visit used to derive low risk of PTB including maternal age, pre-existing diabetes, pre-existing hypertension, other pre-existing maternal medical conditions, asthma, smoking during pregnancy, ethnicity (Aboriginal, Caucasian, other), socioeconomic status in the lowest two quintiles, IVF conception, history of stillbirth, history of PTB and caesarean at last delivery. Rates that were significantly different from 2019 after adjustments for maternal characteristics are shown by ↓ for lower rates and ↑ for higher rates relative to the 2019 rates

For non-Aboriginal women at the tertiary centre, there was a significant decrease in the rate of medically initiated PTB in every year following 2013 (12.4%), reaching its lowest rate in 2015 (8.5%, *p* < 0.001), and the second lowest in 2019 (9.6%, *p* < 0.001). The decrease from 9.1% in 2013 was also statistically significant in the 32–36 week gestation group for every year of the Initiative, with the lowest rate occurring in 2015 (6.0%, *p* < 0.001) followed by 2019 (6.9%, *p* < 0.001). Run charts by gestational age group demonstrated a reduction in PTB overall and in both gestational age groups, for all years of the initiative (Supplementary Fig. 3). For non-Aboriginal women at the tertiary centre, there was also a significant decrease in the spontaneous PTB rate compared to 2013 (7.6%) in 2016 (6.6%, *p* = 0.036), 2017 (5.8%, *p* < 0.001) and 2019 (5.2%, *p* < 0.001). Run charts by gestational age group demonstrated a significant reduction in PTB overall and between 32 and 36 weeks from 2015 to 2019, and in PTB between 20 and 31 weeks from 2017 to 2018 (Supplementary Fig. 3).

For non-Aboriginal women at non-tertiary hospitals, there was a significant increase in the rate of medically initiated PTB in every year following the introduction of the Initiative, from 2.0% in 2013 to a peak of 2.8% in 2018 (*p* < 0.001). Run charts by gestational age group demonstrated a significant increase in the incidence of PTB among births between 20 and 31 weeks in year 2018 and 2019 and overall and between 32 and 36 weeks in years 2015–2019 (Supplementary Fig. 3). For non-Aboriginal women at non-tertiary hospitals the spontaneous PTB rate decreased from 2.5% in 2013, to 2.2% in 2014 (*p* = 0.017), to 2.2% in 2015 (*p* = 0.044), to 2.1% in 2018 (*p* = 0.022), and to 2.2% in 2019 (*p* = 0.039). Run charts by gestational age group demonstrated a significant reduction in the incidence of spontaneous PTB overall and between 32 and 36 weeks in 2014 and 2015, and in 2018 and 2019 (Supplementary Fig. 3).

For Aboriginal women at the tertiary centre, the medically initiated PTB rate in 2013 was 18.0% and in subsequent years was lowest in 2015 (12.7%, *p* = 0.031) and highest in 2017 (19.2%, *p* = 0.637). Run charts by gestational age group demonstrated a reduction in the incidence of medically initiated PTB among births between 32 and 36 weeks during 2014–2016 and 2018–2019, and a reduction overall during 2015 and 2016 (Supplementary Fig. 4). For Aboriginal women at the tertiary centre, the spontaneous PTB rate was 12.9% in 2013 and then ranged from a minimum of 12.8% in 2014 (*p* = 0.946) to a maximum of 18.3% in 2015 (*p* = 0.070). Run charts by gestational age group demonstrated an increase in PTB overall during 2018 and 2019, and in births between 32 and 36 weeks in 2015 (Supplementary Fig. 4).

For Aboriginal women at non-tertiary hospitals, the rate of medically initiated PTB was 2.0% in 2013 and in subsequent years ranged from a minimum of 1.9% in 2015 (*p* = 0.742) to its highest level of 3.6% in 2019 (*p* = 0.020), the most recent year of our evaluation. Run charts by gestational age group demonstrated increases in the incidence of medically initiated PTB overall and in births between 32 and 36 weeks from 2016 (Supplementary Fig. 4). For Aboriginal women at non-tertiary hospitals, the rate of spontaneous PTB was 7.2% in 2013, decreasing to a lowest rate of 5.5% in 2017 (*p* = 0.062) and second lowest rate of 5.8% in 2019 (*p* = 0.147). Run charts by gestational age group demonstrated a reduction in the incidence of spontaneous PTB overall in 2018 and 2019, and in births between 32 and 36 weeks from 2017 (Supplementary Fig. 4).

### Rates of stillbirth

Table 8 shows the annual rates of stillbirth among Aboriginal and non-Aboriginal women, in the tertiary centre, non-tertiary hospitals combined and state-wide from 2013 to 2019, while Supplementary Table 3 shows the adjusted and unadjusted odds of stillbirth compared to 2019.

**Table 8:** Rates of stillbirth per 1000 singleton births, by year ethnicity and hospital, 2013–2019

	Non-aboriginal or Torres Strait Islander women			Aboriginal and Torres Strait Islander women		
	*N*	*n*	per 1000	*N*	*n*	per 1000
**Tertiary centre**						
2013	5059	76	15.02	410	5	12.20
2014	5098	81	15.89	392	7	17.86
2015	4967	76	15.30	361	< 5	< 13.85
2016	4941	69	13.96	380	5	13.16
2017	5103	81	15.87	**365**	**< 5**	**< 13.70↓**
2018	4870	78	16.02	401	12	29.93
2019	5028	72	14.32	377	8	21.22
**Non-tertiary centres**						
2013	26,580	52	1.96	1361	11	8.08
2014	27,131	68	2.51	1394	15	10.76
2015	25,132	64	2.55	**1293**	**5**	**3.87↓**
2016	25,480	71	2.79	1343	16	11.91
2017	23,668	69	2.92	1319	9	6.82
2018	22,774	49	2.15	1261	7	5.55
2019	22,508	53	2.35	1314	16	12.18
**State**						
2013	31,639	128	4.05	1771	16	9.03
2014	32,337	149	4.61	1792	22	12.28
2015	32,213	150	4.66	**1736**	**10**	**5.76↓**
2016	33,048	146	4.42	1823	21	11.52
2017	31,662	160	5.05	**1788**	**11**	**6.15↓**
2018	30,706	133	4.33	1780	22	12.36
2019	30,471	139	4.56	1801	24	13.33

− 470 terminations performed between 20 and 24 pregnancy weeks at the established tertiary centre were excluded (47, 52, 45, 37, 44, 45, 46, 42, 42, 42, 28 in the respective years from 2013 to 2019)

-Stillbirth rates are compared using logistic regression analysis with year 2019 as a reference, univariately and after adjustment for maternal age, nulliparity, grand-multiparty, ethnicity, smoking during pregnancy, maternal asthma, low socioeconomic status, history of stillbirth, placental abruption, antepartum haemorrhage for reasons other than placental abruptions and placenta praevia, gestational diabetes, pre-existing diabetes or hypertension, threatened abortion, threatened preterm labour, IVF conception, caesarean at last delivery and history of PTB. Rates that were significantly different from 2019, both univariately and with adjustments for maternal characteristics are shown by ↓ for lower rates and ↑ for higher rates relative to the 2019 rates

-Stillbirth numbers were suppressed when < 5 cases occurred and the upper bounds for these rates were calculated based on 5 cases

Across the study period stillbirth rates were higher amongst Aboriginal women (11.4 per 1000) compared to non-Aboriginal women (4.7 per 1000). Annual variations were much greater amongst Aboriginal women than amongst non-Aboriginal women due to these women having a lower number of births.

There was no significant change in the stillbirth rate amongst non-Aboriginal women who gave birth at the tertiary centre, following the introduction of the Initiative. The stillbirth rate amongst non-Aboriginal women who gave birth at non-tertiary centres was significantly higher in 2017 compared to 2013 (1.96 per 1000 in 2013 vs 2.92 per 1000 in 2017, *p* = 0.039). The change in the stillbirth rate cannot be explained by any increase in maternal characteristics or obstetric risk factors. There was no significant change in the stillbirth rate amongst non-Aboriginal women who gave birth across the state.

Amongst Aboriginal women who gave birth at the tertiary centre, the stillbirth rate was significantly higher in 2018, when compared to 2013 (12.2 per 1000 in 2013 vs 29.9 per 1000 in 2018, *p* = 0.042). Amongst these women, the maternal risk factor that was higher in 2018 than previous years was caesarean at last birth (18.5% in 2013 vs 25.9% in 2018). Obstetric complications that were slightly higher in 2018 than previous years were threatened preterm labour (10.5% in 2013 vs 12.5% in 2018) and gestational diabetes (11.2% in 2013 vs 14.5% in 2018), although this variation may be able to be explained by small numbers. Examination of the gestational age at which the stillbirth occurred revealed that almost all of these stillbirths occurred at early gestations. Amongst Aboriginal women who gave birth at non-tertiary centres or across the state overall there was no significant change in the stillbirth rate following the introduction of the Initiative.

## Discussion

The current study is the first to examine the effect of the Initiative on PTB amongst Aboriginal women specifically, for whom the PTB rate is higher than the population average. Amongst Aboriginal women there was a small, non- significant reduction in the state-wide PTB rate in the first three years of the Initiative. However, the rate of PTB amongst Aboriginal women has been above pre-initiative (2013) levels since 2017, and reached its highest level in the most recent year of available data (2019). A similar reduction in PTB was observed amongst Aboriginal women giving birth at the tertiary centre in the first 4 years of the Initiative, but this reduction was also not statistically significant, and not maintained to the end of the study period. Amongst non-Aboriginal women, the significant reduction in PTB following the introduction of the WA Initiative has been maintained and improved upon at the tertiary centre, but in non-tertiary hospitals the rate of PTB has increased in recent years.

### Changes in the PTB rate amongst non-Aboriginal women

Amongst non-Aboriginal women, the reduction in PTB at the tertiary centre occurred overall and in the 32–36 weeks gestational age group, with run charts showing a decrease in all years, in all gestational age groups. The initial reduction has not only been maintained, but has been improved upon, with PTB rates in all gestational age groups reaching their lowest incidence since the introduction of the Initiative, in 2019. The reduction among births between 20 and 31 weeks was moderate and is likely due to the introduction of routine cervix length screening at mid-pregnancy morphology scans, followed by vaginal progesterone for women with a shortened cervix or a history of PTB. The major reduction occurred in births between 32 and 36 weeks which may have additionally benefited from a decline in unnecessary iatrogenic delivery, which has resulted from a persistent cultural change amongst clinicians working within the hospital. This hypothesis is consistent with the result that the reductions occurred in women at both low and high risk of PTB, but these reductions were greater in women at low risk amongst whom early iatrogenic delivery is less likely to be required. It is further supported by the fact that births following both spontaneous and medically initiated onset or labour were reduced in this gestational age group, indicating that all of the aforementioned measures likely had an impact.

In contrast to the results observed at the tertiary centre, the PTB rate has risen sharply amongst non-Aboriginal women who gave birth at non-tertiary hospitals in WA, particularly between 32 and 36 weeks. This increase has been driven entirely by iatrogenic PTB, and the increase has been greater in women at high risk of PTB. The Australian Preterm Birth Prevention Alliance observed a similar increase in planned birth, as well as a trend toward these births occurring earlier in pregnancy, with elective caesarean sections performed between 34 and 36 week’s being associated with severe newborn morbidity [11]. Furthermore, New South Wales data from years 2001–2009 showed an increase in planned birth before the due date and hypothesised that the rise in planned births may be due to clinicians taking an increasingly conservative approach for women with pre-labour rupture of membranes, diabetes, low-lying placenta and advanced maternal age [12]. They also concluded that the increase could be driven by clinicians perceiving less risk associated with early delivery due to advancements in neonatal care [12]. Early delivery is indicated where the risks of continuing the pregnancy outweigh the risks associated with prematurity [13], and examination of rates of maternal conditions amongst non-Aboriginal women giving birth at non-tertiary hospitals showed an increase across the study period in advanced maternal age, other pre-existing medical conditions, gestational diabetes and preterm pre-labour rupture of membranes followed by delivery < 37 weeks. However, there were also decreases in rates of smoking and hypertension and these changes in maternal conditions cannot explain the increase in iatrogenic deliveries before 37 weeks gestation.

### Changes in the PTB rate amongst Aboriginal women

Overall, no reduction in PTB rates was observed amongst Aboriginal women giving birth at the tertiary hospital, for whom the PTB rate followed a slowly increasing trend with a sharp rise in 2019. The rise occurred in both the 20–31 and 32–36 week gestational age groups. This increase in the PTB rate that occurred between 2018 and 2019 may be partially due to the observed increase in the rates of pre-eclampsia (6.0% in 2018, 7.4% in 2019) and preterm pre-labour rupture of membranes followed by delivery < 37 weeks (8.0% in 2018, 10.9% in 2019) between 2018 and 2019.

As the major tertiary maternity hospital in the state, it is possible that a large proportion of the preterm births occurring at this hospital were to women who were transferred just prior to the birth of their baby, after the possibility of prevention of the PTB had passed. Compared to non-Aboriginal women, a larger proportion of Aboriginal women live remotely and therefore a disproportionately large number of them may have transferred to the tertiary hospital just prior to birth. Unfortunately, data on where women were receiving their antenatal care prior to the birth of their baby are unavailable so this explanation for the lack of improvement in the PTB rate among Aboriginal women who gave birth at the tertiary hospital cannot be explored. Furthermore, a high proportion of Aboriginal women birthing at the tertiary hospital have risk factors for PTB such as smoking, diabetes and a history of negative obstetric outcomes, with 91.4% of the Aboriginal women who gave birth at the tertiary centre being deemed at high risk of PTB, compared to only 25.1% of non-Aboriginal women. While it may be interpreted as a positive sign that in association with reduced smoking rates, the proportion of high risk women decreased across the study period, unfortunately, this did not translate to a reduction in risk factors for PTB occurring later in pregnancy. It seems more likely that, as was demonstrated with reporting of birth defects in Aboriginal women [14], ascertainment of risk factors is poor amongst Aboriginal women and therefore risk classification lacks the precision to be of use.

Interestingly, the increase in PTB amongst Aboriginal women at the tertiary hospital occurred in preterm births of spontaneous onset indicating that measures such as cervix length measurement, prescription of progesterone and prevention of infection may have been ineffective in this group. This could also be due to a larger proportion of Aboriginal women living outside of the metropolitan region and therefore having less access to mid-pregnancy scans and antenatal care to identify shortened cervix and potential infections. Furthermore, the cost of progesterone as a preventative measure may have been prohibitive for women living in areas assigned lower socioeconomic status, where Aboriginal women are over-represented. In hot weather progesterone also needs to be refrigerated which could pose an additional challenge in the heat of northern Western Australia. Conversely, until 2019, iatrogenic PTB amongst Aboriginal women giving birth at the tertiary hospital was lower than pre-intervention rates, and run chart analysis showed reductions in the 32–36 week age group through 2014–2016 and again in 2018–2019. These results indicate that the cultural change preventing unnecessary medically initiated births that was so effective in non-Aboriginal women at the tertiary hospital, may in fact also have had some impact on the timing of birth amongst Aboriginal women.

Outside of the tertiary centre there appeared to be a small non-significant reduction in PTB amongst Aboriginal women in the four years following the introduction of the Initiative. However, in 2018 and 2019 PTB rates were slightly elevated from pre-Initiative rates. Unlike the results from the tertiary centre, the increases in recent years were driven by an increase in medically initiated births between 32 and 36 weeks. This increase in medically initiated late PTB was similar to that observed at non-tertiary hospitals among non-Aboriginal women. At a state level, the results indicate that the interventions of the Initiative had minimal impact on Aboriginal women, with the exception of the prevention of some late iatrogenic PTB at the tertiary centre, likely resulting from the aforementioned cultural shift at this hospital.

The question remains as to why the Initiative was less effective amongst Aboriginal women than amongst other women giving birth in WA. In a study of Aboriginal women giving birth in Perth, a history of maternal hypertension, vaginal bleeding and consumption of excess alcohol were identified as independent predictors of low birth weight or PTB, with maternal education and smoking also being important risk factors [14]. While smoking cessation campaigns have demonstrated success in the broader population, focus groups have revealed that due to social and economic pressures, smoking cessation is not a priority for many pregnant Indigenous women [15]. Other publications have identified the higher rates of inadequate antenatal care [16] and socioeconomic disadvantage [17] as contributing to the higher rates of PTB in Indigenous women.

Data from New South Wales showed that with extensive efforts from government and healthcare providers, maternal smoking rates declined substantially from 1994 to 2007 across the state. However, the reduction amongst Aboriginal women was minimal and was strongly linked to socioeconomic position [18]. While a reasonable reduction in smoking rates was observed in Aboriginal women across the study period, this reduction was minimal amongst Aboriginal women who gave birth at the tertiary hospital, where the reductions in PTB were observed amongst non-Aboriginal women. This discordance in the response to public health messaging could explain the lack of uniform effect of the Initiative, particularly at the tertiary hospital and highlights the need for specific strategies targeting PTB in Aboriginal women. Community-led programs have shown promise in reducing reported alcohol use during pregnancy [19], while services co-designed with Aboriginal Community Controlled Health such as ‘Birthing on Country’ have been shown to reduce PTB rates in community trials [20]. It is positive that the reduction in medically initiated late PTB observed at the tertiary hospital was also beneficial to Aboriginal women and their babies. However, the outcomes across the state demonstrate that appropriately designed and culturally sensitive programs such as midwifery continuity of care models with Aboriginal representation will be required to improve perinatal outcomes more broadly, and that more targeted and collaborative approaches must be employed.

### Implementation of the initiative at non-tertiary hospitals

In non-tertiary hospitals the PTB rate rose amongst both Aboriginal and non-Aboriginal women, primarily driven by an increase in iatrogenic late PTB. Unfortunately, despite the increased prematurity aimed at reducing the chances of stillbirth, there was no reduction in the stillbirth rate at non-tertiary hospitals. The success in WA at reducing PTB rates at the tertiary hospital demonstrates that we have the knowledge and capability to lower the rate of PTB. The factor limiting effectiveness is the implementation of these strategies in the non-tertiary environment. The recipe to change culture and make it happen in the non-tertiary environment requires a sustained and collaborative model, with ongoing engagement and advocacy, such as the work that the Australian Preterm Birth Prevention Alliance engages in. As a result of their efforts, and in recognition of the disparity in results between the tertiary and non-tertiary hospitals, the lessons of the Initiative have now lead to embracing additional methodologies funded by the federal government, based on the breakthrough collaborative model (BTS). In the coming years, 52 hospitals from around Australia will participate in the BTS, a program which will bring together teams seeking reductions in PTB based on existing best practice already implemented in the tertiary hospital. The BTS model has demonstrated previous success in improving quality healthcare, including in the maternity environment with substantial reductions in caesarean section rates being observed in participating hospitals following the implementation of the model [21]. In time this model may be used to reduce the PTB rate in the non-tertiary environment.

### Changes in the stillbirth rate

The higher stillbirth rate amongst Aboriginal women compared to non-Aboriginal women is likely due to their higher burden of obstetric conditions and pre-existing maternal conditions. While there was some variation in the stillbirth rate between years, at the tertiary hospital where the major reductions in PTB were observed amongst non-Aboriginal women, there was no associated increase in the stillbirth rate.

Increases in the stillbirth rate were observed amongst Aboriginal women giving birth at the tertiary hospital in 2018, and amongst non-Aboriginal women giving birth at non-tertiary hospitals in 2017. Compared to the years prior, neither of these increases were associated with a significant reduction in medically initiated preterm births or the overall preterm birth rate, and therefore are unlikely to be associated with any interventions of the Initiative. Furthermore, the stillbirths amongst Aboriginal women at the tertiary centre in 2018 were almost entirely in the 20–31 week gestational age group, indicating that they could not have resulted from delaying medically indicated deliveries. Further investigations did not reveal any major increases in risk factors in these years that could explain the elevated rates.

### Strengths and limitations

The size of the study population was the major strength of this study, with 11 years of data for the whole state being included. Our large sample allowed us to separately investigate the impact of the Initiative on PTB rates amongst Aboriginal women for the first time. It also allowed us to conduct subgroup analyses by hospital, gestational age group, onset of labour and risk of PTB, which revealed valuable insights which may otherwise have been concealed. The use of a population-based prospective cohort study design based on administrative data is appropriate, but comes with the limitation of a lack of concurrent controls which could introduce bias. Our introduction of low and high risk of PTB status mitigates this limitation. Another limitation is that the ethnicity variables used to derive Aboriginal and Torres Strait Islander ethnicity are known to be under-reported in population-based health and health related data collections [22]. It is also generally accepted that ascertainment of risk factors amongst this group is less robust. This source of bias was minimised by removing the inconsistencies and assigning Aboriginal status when the majority of a woman’s pregnancies were reported as Aboriginal ethnicity. Finally, previous analyses have shown that rates of PTB were higher in private hospitals [8]. In this study we were not able to separate our analysis of non-tertiary hospitals into private and public, which may have concealed differences between these two types of hospitals.

## Conclusions

The successful and sustained reduction in PTB amongst non-Aboriginal women at the tertiary hospital demonstrates that lowering the PTB rate is possible, and the challenge is to successfully implement these strategies in the non-tertiary environment. The major gains were made in low risk pregnancies, and affected PTB of both spontaneous and non-spontaneous onset. While reductions in medically initiated PTB were also observed amongst Aboriginal women at the tertiary hospital, across the state, the interventions of the Initiative had minimal impact on Aboriginal women with the PTB rate rising above pre-Initiative levels from 2017. Design of more appropriate and culturally responsive programs will be required to lower the PTB rate amongst these women.

## Supplementary Information

**Additional file 1: Supplementary Table 1.** Rates of preterm birth for maternal characteristics known at the time of the first antenatal visit. **Supplementary Table 2**. Rates of PTB for maternal characteristics and pregnancy complications, by ethnicity and risk (2009–2019). **Supplementary Table 3**. Rates of stillbirth per 1000 singleton births, by year ethnicity and hospital.

**Additional file 2: Supplementary Fig. 1.** Rates of preterm birth in singleton pregnancies, by gestational age, hospital level and risk, non-Aboriginal or Torres Strait Islander Women.

**Additional file 3: Supplementary Fig. 2.** Rates of preterm birth in singleton pregnancies, by gestational age, hospital level and risk, Aboriginal women.

**Additional file 4: Supplementary Fig. 3.** Rates of preterm birth in singleton pregnancies, by gestational age, hospital level and onset, non-Aboriginal or Torres Strait Islander Women.

**Additional file 5: Supplementary Fig. 4.** Rates of preterm birth in singleton pregnancies, by gestational age, hospital level and onset, Aboriginal women.

## Data Availability

The authors do not have permission to share patient-level data extracted from the Data Linkage Unit of the Department of Health of Western Australia. Data can only be made available to researchers who apply to the Department of Health of Western Australia’s Human Research Ethics Committee (https://ww2.health.wa.gov.au/Articles/A_E/Department-of-Health-Human-Research-Ethics-Committee) and Data Linkage Unit (www.datalinkage-wa.org.au). Please contact the lead author, Ye’elah Berman (yeelah.berman@uwa.edu.au) to discuss the availability of data further.

## Abbreviations

PTB: Preterm birth
WA: Western Australia
MNS: Midwives Notification System
IVF: In-vitro fertilization
BTS: Breakthrough series
