# Supplementary material for: The Western Australian preterm birth prevention initiative: a whole of state singleton pregnancy cohort study showing the need to embrace alternative models of care for Aboriginal women
Source: BMC Pregnancy Childbirth. 2023 Jan 4;23:7. doi: 10.1186/s12884-022-05222-9 (PMC9811788; doi:10.1186/s12884-022-05222-9)
Supplement: Supplementary file 1 — Additional file 1: Supplementary Table 1. Rates of preterm birth for maternal characteristics known at the time of the first antenatal visit. Supplementary Table 2. Rates of PTB for maternal characteristics and pregnancy complications, by ethnicity and risk (2009–2019). Supplementary Table 3. Rates of stillbirth per 1000 singleton births, by year ethnicity and hospital. [file 12884_2022_5222_MOESM1_ESM.docx]

**Supplementary Table 1. Rates of preterm birth for maternal characteristics known at the time of the first antenatal visit**

|  |  | **PTB (%)** | **Low Risk**  **PTB (%)** | **High Risk**  **PTB (%)** | **OR (95%CI)** | **p** |
| --- | --- | --- | --- | --- | --- | --- |
| **Maternal demographics** |  |  |  |  |  |  |
| Maternal age | 20-34 | 6.8 | 5.5 | 14.8 | 1.00 | - |
|  | <20 | 10.4 | 8.0 | 11.6 | 1.58 (1.48, 1.68) | <.001 |
|  | ≥35 | 8.0 | 5.7 | 12.8 | 1.19 (1.15, 1.22) | <.001 |
| Ethnicity | Caucasian | 6.7 | 5.4 | 13.4 | 1.00 | - |
|  | Other | 7.0 | 5.9 | 12.9 | 1.05 (1.02, 1.08) | 0.002 |
|  | Indigenous | 14.1 | 8.4 | 15.3 | 2.28 (2.19, 2.38) | <.001 |
| Parity | 1-4 | 6.8 | 5.0 | 16.5 | 1.00 | - |
|  | ≥5 | 13.1 | 7.2 | 19.4 | 2.06 (1.91, 2.24) | <.001 |
|  | 0 | 7.5 | 6.4 | 10.9 | 1.11 (1.08, 1.14) | <.001 |
| Smoking during pregnancy | No | 6.6 | 5.5 | 14.0 | 1.00 | - |
|  | Yes | 12.0 | 7.5 | 13.3 | 1.92 (1.86, 1.99) | <.001 |
| Socio-Economic Indexes for Areas (SEIFA) in Lowest 40% | No | 7.0 | 5.6 | 13.6 | 1.00 | - |
|  | Yes | 8.1 | 5.6 | 14.4 | 1.17 (1.13, 1.2) | <.001 |
|  | Unknown | 7.3 | 6.3 | 9.3 | 1.05 (0.91, 1.21) | <.001 |
| **Medical Conditions** |  |  |  |  |  |  |
| Pre-existing diabetes | No | 7.0 | 5.6 | 13.0 | 1.00 | - |
|  | Yes | 31.2 |  | 31.2 | 6.02 (5.55, 6.53) | <.001 |
| Pre-existing hypertension | No | 7.1 | 5.6 | 13.4 | 1.00 | - |
|  | Yes | 19.2 | 8.3 | 19.6 | 3.12 (2.88, 3.39) | <.001 |
| Asthma | No | 7.1 | 5.5 | 13.8 | 1.00 | - |
|  | Yes | 8.0 | 6.0 | 13.4 | 1.14 (1.09, 1.19) | <.001 |
| Other pre-existing conditions | No | 6.5 | 5.3 | 13.4 | 1.00 | - |
|  | Yes | 8.5 | 6.1 | 14.1 | 1.32 (1.29, 1.36) | <.001 |
| **Obstetric history** |  |  |  |  |  |  |
| Previous PTB^+^ | No | 4.6 | 4.3 | 11.5 | 1.00 | - |
|  | Yes | 25.3 |  | 25.3 | 7.08 (6.72, 7.47) | <.001 |
|  | Unknown | 6.9 | 5.7 | 12.7 | 1.56 (1.5, 1.62) | <.001 |
| Previous stillbirths | No | 7.0 | 5.5 | 13.4 | 1.00 | - |
|  | 1 | 18.2 | 11.3 | 20.8 | 2.94 (2.72, 3.19) | <.001 |
|  | ≥2 | 22.9 | 14.3 | 23.5 | 3.91 (3, 5.09) | <.001 |
| Caesarean at last birth | No | 7.0 | 5.5 | 13.0 | 1.00 | - |
|  | Yes | 8.2 | 5.9 | 17.1 | 1.2 (1.16, 1.24) | <.001 |
| IVF conception | No | 7.1 | 5.6 | 14.0 | 1.00 | - |
|  | Yes | 10.3 | 6.1 | 11.7 | 1.51 (1.42, 1.60) | <.001 |

-nulliparous and parous women combined after predictive models for nulliparous (n=152418) and parous (n=206889) women were constructed separately.

-singleton births from 2009-2019 included

-OR (95%CI)=unadjusted odds ratio and confidence interval, PTB (%) = PTB incidence rate

^+^ nulliparous women removed from these rates

**Supplementary Table 2. Rates of PTB for maternal characteristics and pregnancy complications, by ethnicity and risk (2009-2019)**

|  |  | **Non-Aboriginal or Torres Strait Islander** | | | | **Aboriginal and Torres Strait Islander** | | | |
| --- | --- | --- | --- | --- | --- | --- | --- | --- | --- |
|  |  | **Low Risk** | | **High Risk** | | **Low Risk** | | **High Risk** | |
|  |  | **COL %** | **PTB %** | **COL %** | **PTB %** | **COL %** | **PTB %** | **COL %** | **PTB %** |
| Preterm birth |  | 83.7 | 5.5 | 16.3 | 13.3 | 17.1 | 8.4 | 82.9 | 15.3 |
| Maternal age | 20-34 | 80.2 | 5.4 | 50.8 | 14.6 | 98.8 | 8.4 | 68.3 | 15.3 |
|  | <20 | 1.4 | 8.0 | 7.6 | 10.1 |  |  | 21.8 | 13.4 |
|  | ≥35 | 18.3 | 5.7 | 41.5 | 12.3 | 1.2 | 5.0 | 9.9 | 19.3 |
| Ethnicity | Caucasian | 75.1 | 5.4 | 75.1 | 13.4 |  |  |  |  |
|  | Other | 24.9 | 5.9 | 24.9 | 12.9 |  |  |  |  |
|  | Indigenous |  |  |  |  | 100.0 | 8.4 | 100.0 | 15.3 |
| Parity | 0 | 40.6 | 6.4 | 55.9 | 10.6 |  |  | 36.6 | 12.6 |
|  | 1-4 | 58.4 | 4.9 | 41.9 | 16.5 | 95.2 | 8.4 | 54.6 | 16.5 |
|  | >=5 | 0.9 | 7.3 | 2.3 | 19.8 | 4.8 | 6.9 | 8.8 | 18.9 |
| Smoking during pregnancy | No | 97.0 | 5.5 | 64.1 | 14.1 | 100.0 | 8.4 | 44.3 | 13.6 |
|  | Yes | 3.0 | 7.5 | 35.9 | 11.8 |  |  | 55.7 | 16.7 |
| Socio-Economic Indexes for Areas (SEIFA) in Lowest 40% | No | 83.0 | 5.5 | 79.2 | 13.2 | 51.1 | 9.3 | 49.4 | 15.6 |
|  | Yes | 16.4 | 5.5 | 19.2 | 14.0 | 48.5 | 7.3 | 50.2 | 15.0 |
|  | Unknown | 0.6 | 6.2 | 1.6 | 8.3 | 0.4 | 25.0 | 0.3 | 25.0 |
| **Medical conditions** |  |  |  |  |  |  |  |  |  |
| Pre-existing diabetes | No | 100.0 | 5.5 | 95.9 | 12.6 | 100.0 | 8.4 | 97.0 | 14.5 |
|  | Yes |  |  | 4.1 | 29.3 |  |  | 3.0 | 40.3 |
| Pre-existing hypertension | No | 99.9 | 5.5 | 93.8 | 12.9 | 100.0 | 8.4 | 98.4 | 15.0 |
|  | Yes | 0.1 | 8.3 | 6.2 | 18.4 |  |  | 1.6 | 36.8 |
| Asthma | No | 91.9 | 5.5 | 86.8 | 13.3 | 94.7 | 8.3 | 90.0 | 15.4 |
|  | Yes | 8.1 | 6.0 | 13.2 | 13.2 | 5.3 | 9.1 | 10.0 | 14.0 |
| Other pre-existing conditions | No | 69.6 | 5.3 | 47.2 | 12.9 | 81.5 | 8.8 | 53.9 | 14.9 |
|  | Yes | 30.4 | 6.1 | 52.8 | 13.6 | 18.5 | 6.4 | 46.1 | 15.8 |
| **Obstetric history** |  |  |  |  |  |  |  |  |  |
| Previous PTB | No | 69.0 | 5.5 | 58.7 | 10.7 | 51.6 | 7.7 | 49.5 | 12.2 |
|  | Yes |  |  | 18.0 | 23.8 |  |  | 9.4 | 34.9 |
|  | Unknown | 31.0 | 5.6 | 23.3 | 11.8 | 48.4 | 9.1 | 41.0 | 14.5 |
| Previous stillbirths | 0 | 99.6 | 5.5 | 95.0 | 13.0 | 99.8 | 8.4 | 96.6 | 14.8 |
|  | 1 | 0.4 | 11.3 | 4.6 | 19.3 | 0.2 | 20.0 | 3.1 | 28.4 |
|  | ≥2 | 0.0 | 14.3 | 0.5 | 20.4 |  |  | 0.3 | 40.9 |
| Caesarean at last birth | No | 83.4 | 5.5 | 81.4 | 12.4 | 92.1 | 8.4 | 84.7 | 15.0 |
|  | Yes | 16.6 | 5.8 | 18.6 | 17.2 | 7.9 | 7.6 | 15.3 | 16.8 |
| IVF conception | No | 99.0 | 5.5 | 84.7 | 13.6 | 99.9 | 8.4 | 99.7 | 15.3 |
|  | Yes | 1.0 | 6.1 | 15.3 | 11.7 | 0.1 |  | 0.3 | 5.5 |
| **Complications of pregnancy** |  |  |  |  |  |  |  |  |  |
| Stillbirth | No | 99.6 | 5.2 | 99.3 | 12.8 | 99.5 | 8.0 | 98.7 | 14.4 |
|  | Yes | 0.4 | 79.6 | 0.7 | 84.9 | 0.5 | 72.2 | 1.3 | 84.2 |
| Threatened preterm labour | No | 98.3 | 4.7 | 96.2 | 11.4 | 96.8 | 7.2 | 94.8 | 13.1 |
|  | Yes | 1.7 | 53.1 | 3.8 | 61.6 | 3.2 | 44.8 | 5.2 | 55.8 |
| Gestational Diabetes | No | 92.3 | 5.3 | 89.7 | 13.1 | 92.3 | 8.4 | 92.3 | 15.3 |
|  | Yes | 7.7 | 7.7 | 10.3 | 15.1 | 7.7 | 7.9 | 7.7 | 15.7 |
| Pre-eclampsia | No | 98.2 | 5.1 | 95.9 | 12.0 | 98.7 | 8.0 | 96.7 | 14.4 |
|  | Yes | 1.8 | 30.4 | 4.1 | 44.0 | 1.3 | 34.9 | 3.3 | 42.0 |
| Antepartum Haemorrhage | No | 97.3 | 5.0 | 95.6 | 12.1 | 97.4 | 7.4 | 97.1 | 14.3 |
|  | Yes | 2.7 | 24.1 | 4.4 | 38.9 | 2.6 | 44.7 | 2.9 | 48.2 |
| Preterm pre-labour rupture of membranes with delivery <37 weeks | No | 98.8 | 4.4 | 97.5 | 11.1 | 98.2 | 6.7 | 96.4 | 12.1 |
|  | Yes | 1.2 | 100.0 | 2.5 | 100.0 | 1.8 | 100.0 | 3.6 | 100.0 |

**Supplementary Figure 1. Rates of preterm birth in singleton pregnancies, by gestational age, hospital level and risk, non-Aboriginal or Torres Strait Islander Women**


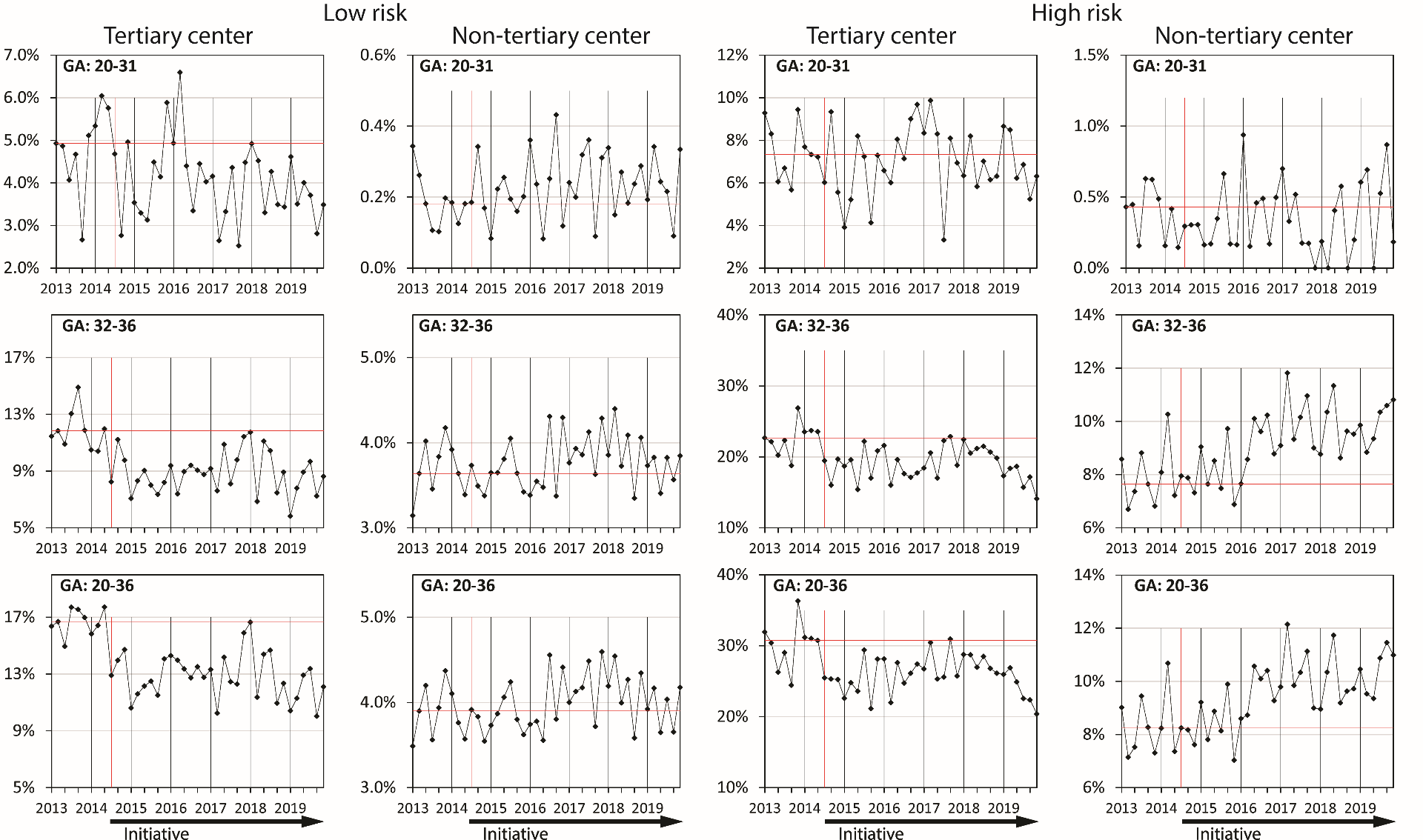


-The minimum and maximum number of births per two month time epoch were: Low risk Tertiary centre: 561, 677, Low risk Non-tertiary centre: 2991, 4095, High risk Tertiary centre: 179, 248, High risk Non-tertiary centre: 467, 721.

**Supplementary Figure 2. Rates of preterm birth in singleton pregnancies, by gestational age, hospital level and risk, Aboriginal women**

**

**

-run charts for the 20-31 weeks gestational age group, and low risk women who gave birth at the tertiary centre have been omitted due to small numbers.

-The minimum and maximum number of births per two month time epoch were: Low risk Non-tertiary centre: 29, 63, High risk Tertiary centre: 41, 76, High risk Non-tertiary centre: 140, 215.

**Supplementary Figure 3. Rates of preterm birth in singleton pregnancies, by gestational age, hospital level and onset, non-Aboriginal or Torres Strait Islander Women**

**
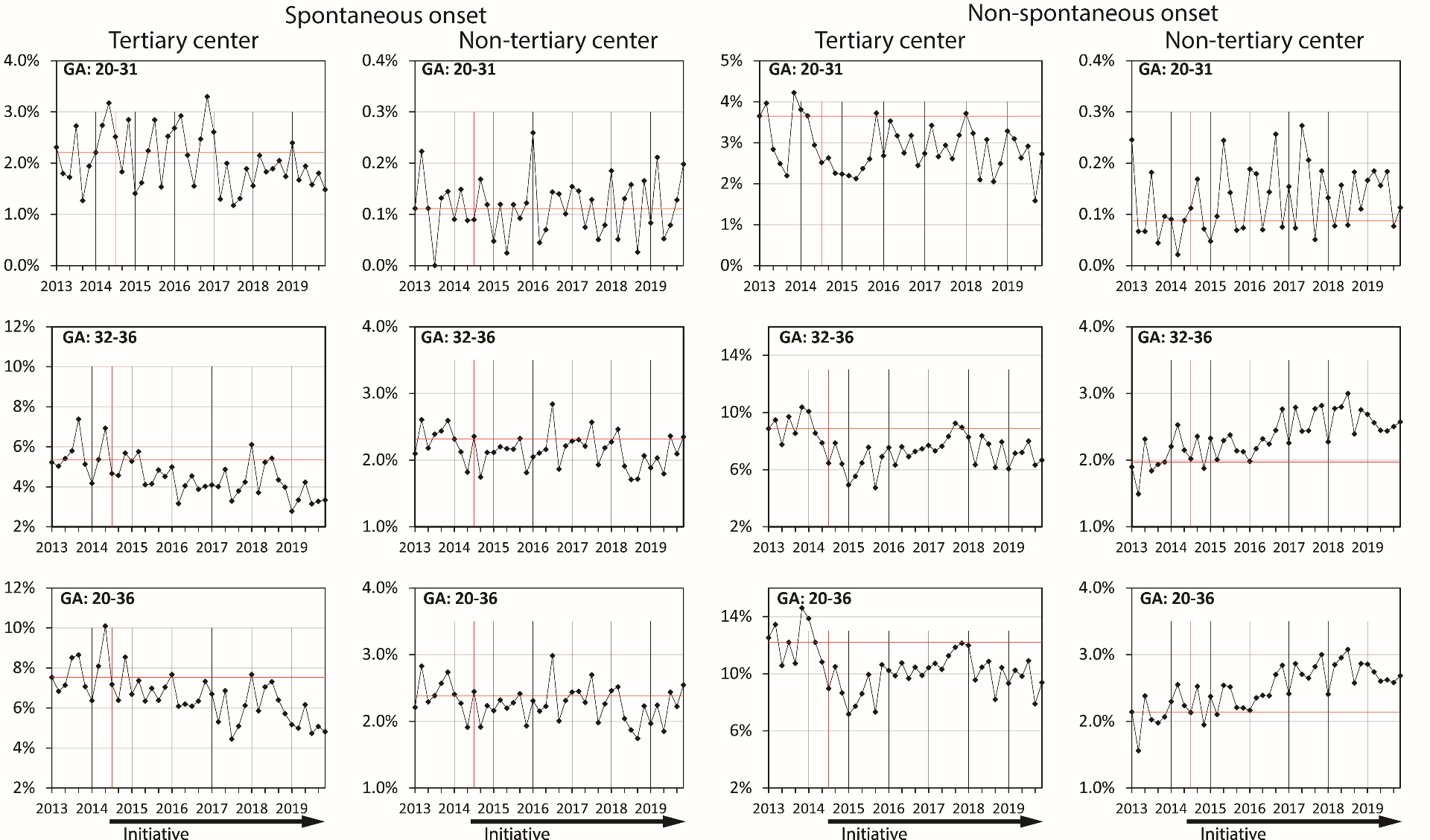
**

-The minimum and maximum number of births per two month time epoch were: Tertiary centre: 753, 903, Non-tertiary centre: 3537, 4755.

**Supplementary Figure 4. Rates of preterm birth in singleton pregnancies, by gestational age, hospital level and onset, Aboriginal women**


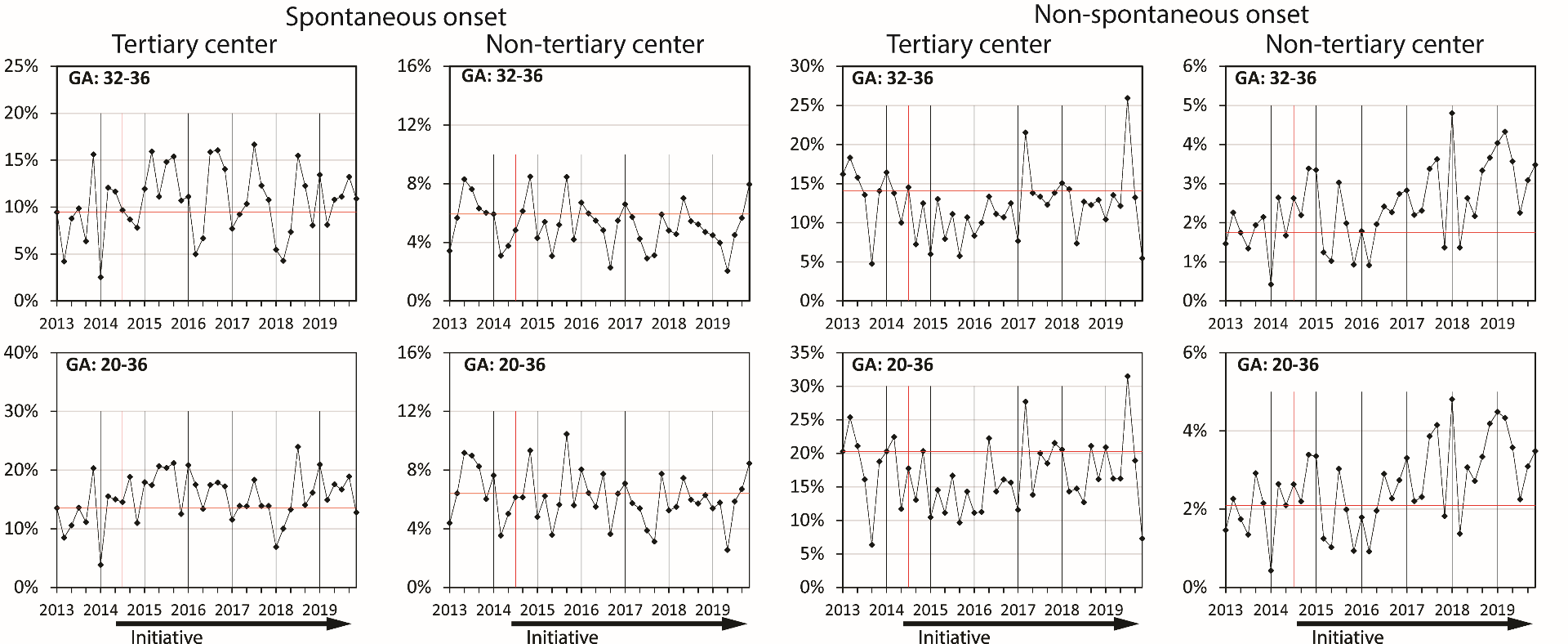


-run charts for the 20-31 weeks gestational age group have been omitted due to small numbers

-The minimum and maximum number of births per two month time epoch were: Tertiary centre: 45, 81, Non-tertiary centre: 184, 277.

**Supplementary Table 3. Rates of stillbirth per 1000 singleton births, by year ethnicity and hospital**

|  | **Non-Aboriginal or Torres Strait Islander Women** | | | | | | | | **Aboriginal and Torres Strait Islander Women** | | | | | | |
| --- | --- | --- | --- | --- | --- | --- | --- | --- | --- | --- | --- | --- | --- | --- | --- |
| **Tertiary centre** | **N** | **n** | **per 1000** | **OR (95% CI)** | **p** | **aOR (95% CI)** | **p** | **N** | | **n** | **per 1000** | **OR (95% CI)** | **p** | **aOR (95% CI)** | **p** |
| **2013** | 5059 | 76 | 15.02 | 1.05 (0.76, 1.45) | 0.769 | 1.05 (0.76, 1.46) | 0.763 | 410 | | 5 | 12.2 | 0.57 (0.19, 1.76) | 0.327 | 0.41 (0.12, 1.34) | 0.14 |
| **2014** | 5098 | 81 | 15.89 | 1.11 (0.81, 1.53) | 0.518 | 1.11 (0.80, 1.54) | 0.53 | 392 | | 7 | 17.86 | 0.84 (0.30, 2.34) | 0.736 | 0.63 (0.21, 1.87) | 0.405 |
| **2015** | 4967 | 76 | 15.3 | 1.07 (0.77, 1.48) | 0.685 | 1.06 (0.77, 1.48) | 0.712 | 361 | | <5 | <13.85 | 0.52 (0.15, 1.73) | 0.285 | 0.48 (0.14, 1.66) | 0.246 |
| **2016** | 4941 | 69 | 13.96 | 0.98 (0.70, 1.36) | 0.881 | 0.93 (0.66, 1.30) | 0.664 | 380 | | 5 | 13.16 | 0.62 (0.20, 1.90) | 0.398 | 0.58 (0.18, 1.84) | 0.354 |
| **2017** | 5103 | 81 | 15.87 | 1.11 (0.81, 1.53) | 0.522 | 1.08 (0.78, 1.49) | 0.639 | 365 | | <5 | <13.70 | 0.25 (0.05, 1.21) | 0.085 | **0.19 (0.04, 0.93)** | **0.041** |
| **2018** | 4870 | 78 | 16.02 | 1.12 (0.81, 1.55) | 0.49 | 1.12 (0.81, 1.55) | 0.497 | 401 | | 12 | 29.93 | 1.42 (0.58, 3.52) | 0.445 | 1.31 (0.52, 3.31) | 0.575 |
| **2019** | 5028 | 72 | 14.32 | ref | - | ref | - | 377 | | 8 | 21.22 | ref | - | ref | - |
| **Non-tertiary centres** | **N** | **n** | **per 1000** | **OR (95% CI)** | **p** | **aOR (95% CI)** | **p** | **N** | | **n** | **per 1000** | **OR (95% CI)** | **p** | **aOR (95% CI)** | **p** |
| **2013** | 26580 | 52 | 1.96 | 0.83 (0.57, 1.22) | 0.342 | 0.81 (0.55, 1.19) | 0.278 | 1361 | | 11 | 8.08 | 0.66 (0.31, 1.43) | 0.293 | 0.68 (0.31, 1.50) | 0.336 |
| **2014** | 27131 | 68 | 2.51 | 1.07 (0.74, 1.53) | 0.733 | 1.04 (0.73, 1.50) | 0.829 | 1394 | | 15 | 10.76 | 0.88 (0.44, 1.79) | 0.729 | 0.94 (0.45, 1.95) | 0.866 |
| **2015** | 25132 | 64 | 2.55 | 1.08 (0.75, 1.56) | 0.673 | 1.07 (0.74, 1.55) | 0.71 | 1293 | | 5 | 3.87 | **0.32 (0.12, 0.86)** | **0.025** | **0.30 (0.11, 0.84)** | **0.022** |
| **2016** | 25480 | 71 | 2.79 | 1.18 (0.83, 1.69) | 0.353 | 1.14 (0.80, 1.63) | 0.469 | 1343 | | 16 | 11.91 | 0.98 (0.49, 1.96) | 0.951 | 0.95 (0.47, 1.94) | 0.888 |
| **2017** | 23668 | 69 | 2.92 | 1.24 (0.87, 1.77) | 0.242 | 1.19 (0.83, 1.71) | 0.339 | 1319 | | 9 | 6.82 | 0.56 (0.25, 1.27) | 0.163 | 0.48 (0.20, 1.12) | 0.09 |
| **2018** | 22774 | 49 | 2.15 | 0.91 (0.62, 1.35) | 0.649 | 0.89 (0.60, 1.31) | 0.551 | 1261 | | 7 | 5.55 | 0.45 (0.19, 1.10) | 0.082 | 0.49 (0.20, 1.21) | 0.122 |
| **2019** | 22508 | 53 | 2.35 | ref | - | ref | - | 1314 | | 16 | 12.18 | ref | - | ref | - |
| **State** | **N** | **n** | **per 1000** | **OR (95% CI)** | **p** | **aOR (95% CI)** | **p** | **N** | | **n** | **per 1000** | **OR (95% CI)** | **p** | **aOR (95% CI)** | **p** |
| **2013** | 31639 | 128 | 4.05 | 0.89 (0.70, 1.13) | 0.326 | 0.87 (0.69, 1.11) | 0.277 | 1771 | | 16 | 9.03 | 0.68 (0.36, 1.28) | 0.226 | 0.62 (0.32, 1.19) | 0.15 |
| **2014** | 32337 | 149 | 4.61 | 1.01 (0.80, 1.27) | 0.932 | 1.00 (0.79, 1.26) | 0.981 | 1792 | | 22 | 12.28 | 0.92 (0.51, 1.65) | 0.78 | 0.85 (0.47, 1.55) | 0.594 |
| **2015** | 32213 | 150 | 4.66 | 1.02 (0.81, 1.29) | 0.861 | 1.02 (0.81, 1.28) | 0.893 | 1736 | | 10 | 5.76 | **0.43 (0.21, 0.90)** | **0.025** | **0.43 (0.20, 0.90)** | **0.026** |
| **2016** | 33048 | 146 | 4.42 | 0.97 (0.77, 1.22) | 0.786 | 0.94 (0.75, 1.19) | 0.612 | 1823 | | 21 | 11.52 | 0.86 (0.48, 1.56) | 0.624 | 0.82 (0.45, 1.50) | 0.521 |
| **2017** | 31662 | 160 | 5.05 | 1.11 (0.88, 1.39) | 0.376 | 1.08 (0.86, 1.36) | 0.502 | 1788 | | 11 | 6.15 | **0.46 (0.22, 0.94)** | **0.033** | **0.38 (0.18, 0.80)** | **0.011** |
| **2018** | 30706 | 133 | 4.33 | 0.95 (0.75, 1.21) | 0.669 | 0.94 (0.74, 1.20) | 0.615 | 1780 | | 22 | 12.36 | 0.93 (0.52, 1.66) | 0.797 | 0.95 (0.53, 1.71) | 0.858 |
| **2019** | 30471 | 139 | 4.56 | ref | - | ref | - | 1801 | | 24 | 13.33 | ref | - | ref | - |

-470 terminations performed between 20–24 pregnancy weeks at the established tertiary centre were excluded (47, 52, 45, 37, 44, 45, 46, 42, 42, 42, 28 in the respective years from 2013 to 2019)

-Stillbirth rates are compared using logistic regression analysis with year 2019 as a reference, univariately and after adjustment for maternal age, nulliparity, grand-multiparty, ethnicity, smoking during pregnancy, maternal asthma, low socioeconomic status, history of stillbirth, placental abruption, antepartum haemorrhage for reasons other than placental abruptions and placenta praevia, gestational diabetes, pre-existing diabetes or hypertension, threatened abortion, threatened preterm labour, IVF conception, caesarean at last delivery and history of PTB. Rates that were significantly different from 2019, both univariately and with adjustments for maternal characteristics are bolded. SB cases were supressed when <5 cases occurred and the upper bounds for these rates were calculated based on 5 cases.
