# Supplementary figures and images for: The Western Australian preterm birth prevention initiative: a whole of state singleton pregnancy cohort study showing the need to embrace alternative models of care for Aboriginal women
Source: BMC Pregnancy Childbirth. 2023 Jan 4;23:7. doi: 10.1186/s12884-022-05222-9 (PMC9811788; doi:10.1186/s12884-022-05222-9)

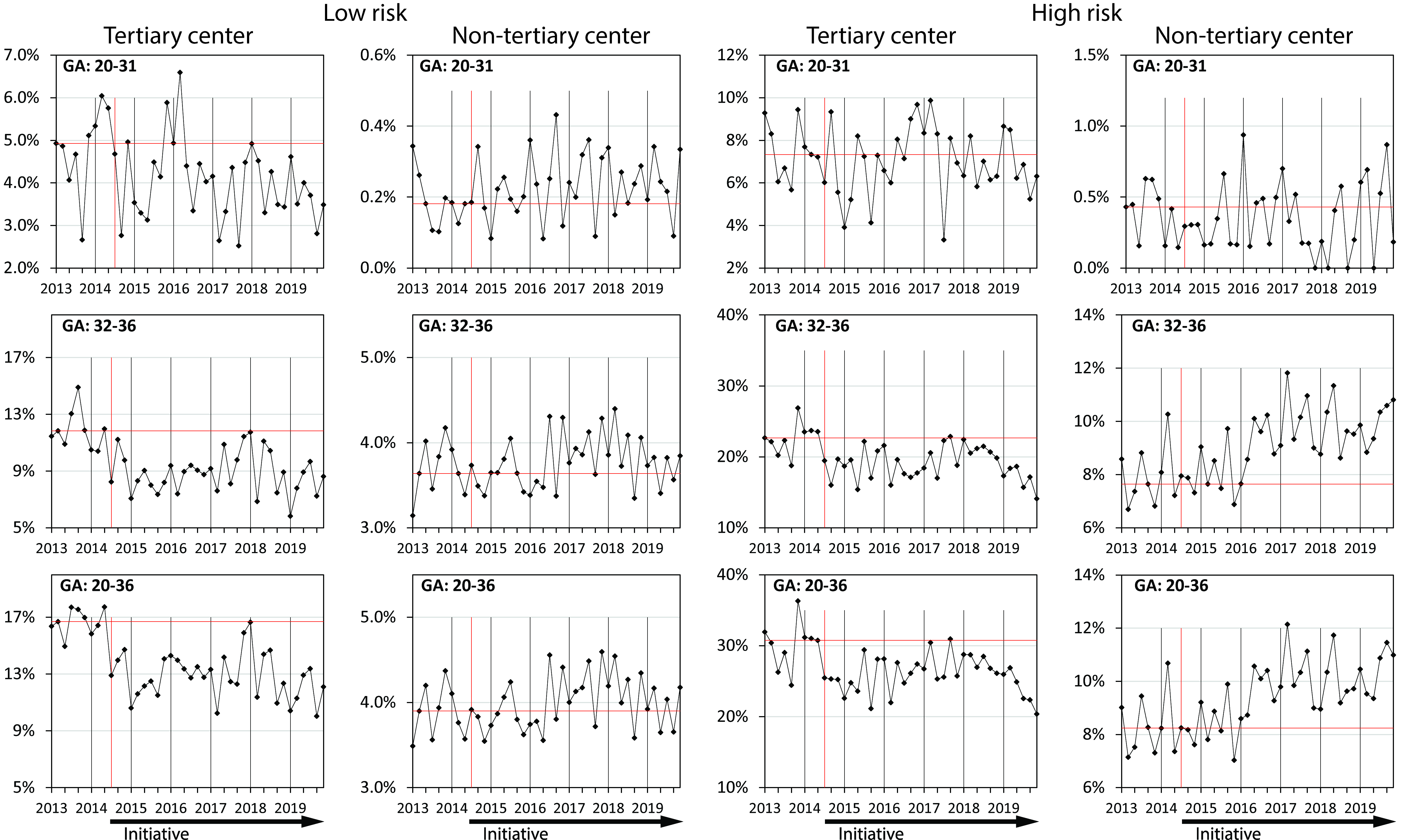

Supplement: Supplementary file 2 — Additional file 2: Supplementary Fig. 1. Rates of preterm birth in singleton pregnancies, by gestational age, hospital level and risk, non-Aboriginal or Torres Strait Islander Women. [file 12884_2022_5222_MOESM2_ESM.tif]

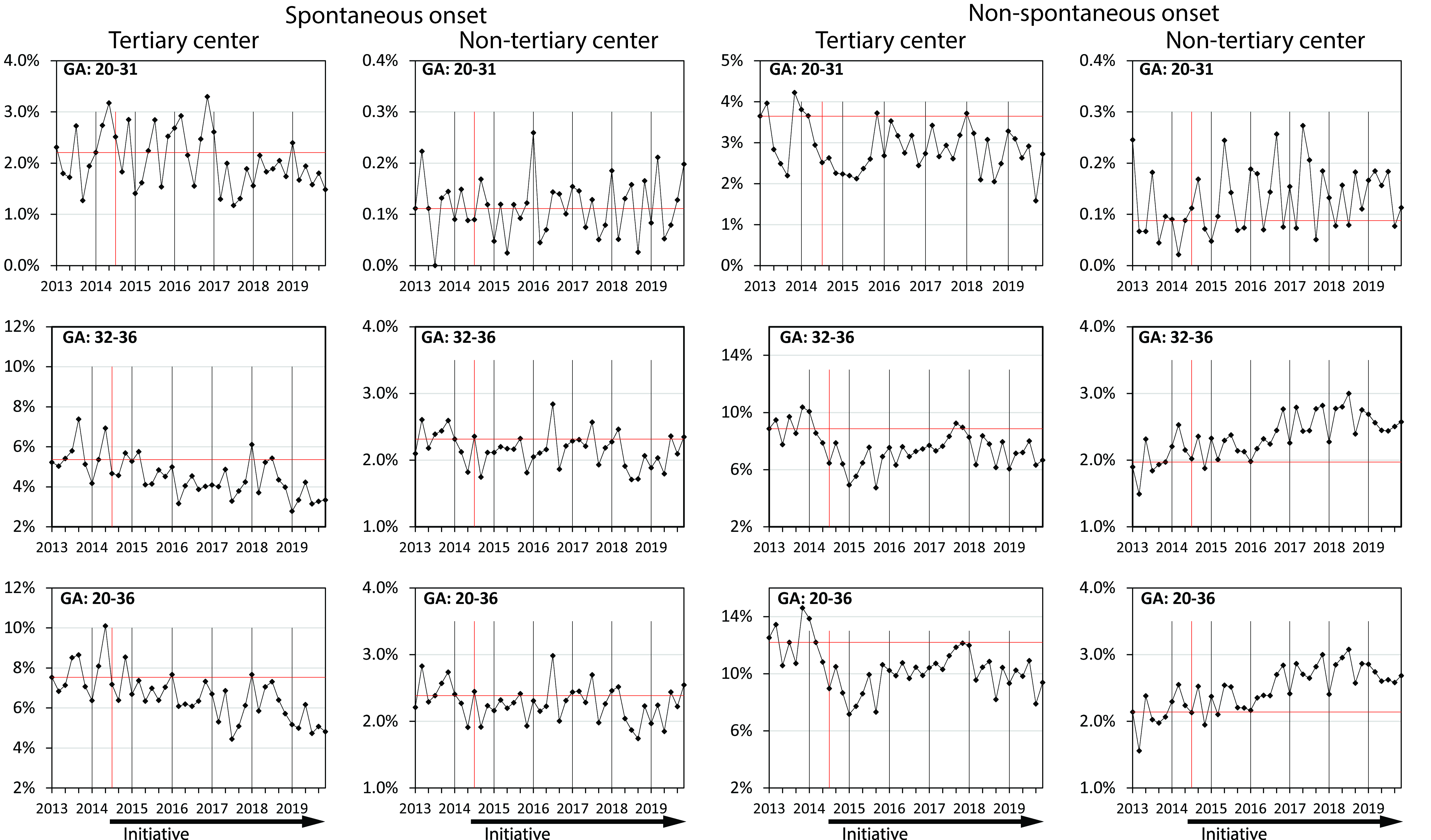

Supplement: Supplementary file 4 — Additional file 4: Supplementary Fig. 3. Rates of preterm birth in singleton pregnancies, by gestational age, hospital level and onset, non-Aboriginal or Torres Strait Islander Women. [file 12884_2022_5222_MOESM4_ESM.tif]

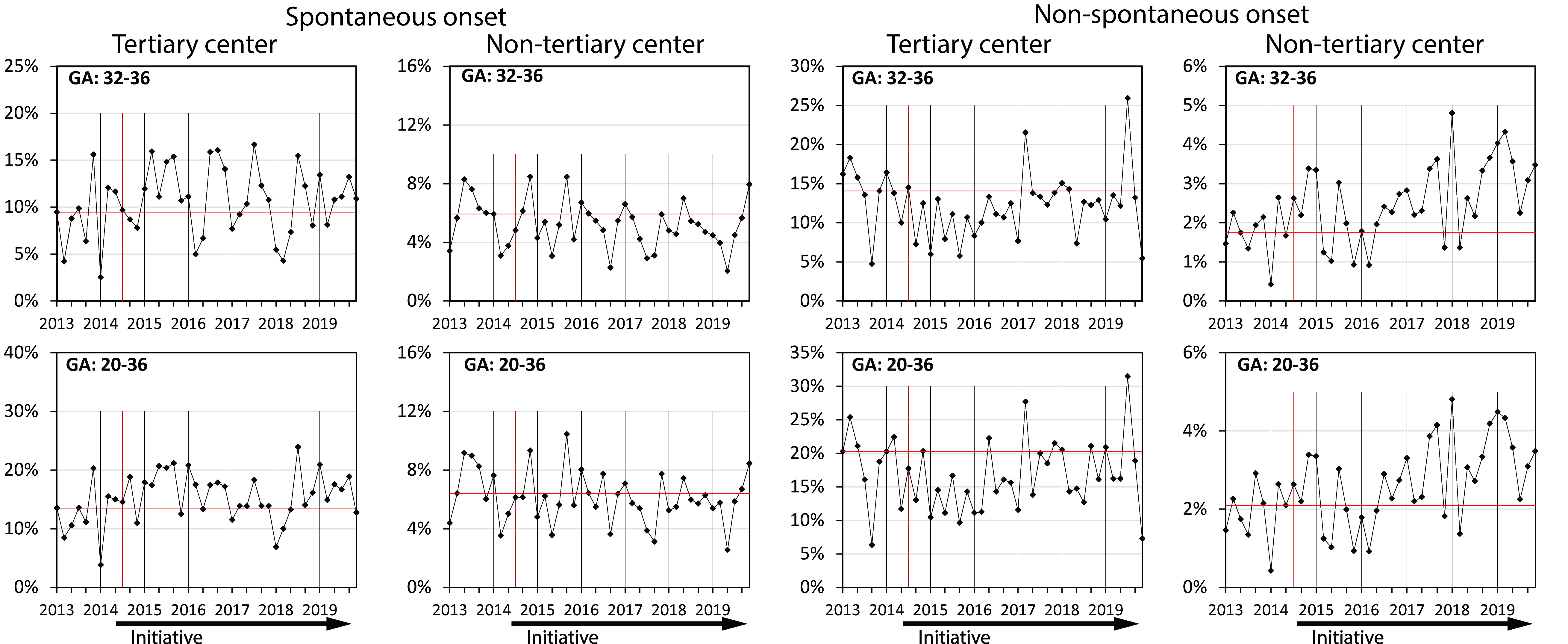

Supplement: Supplementary file 5 — Additional file 5: Supplementary Fig. 4. Rates of preterm birth in singleton pregnancies, by gestational age, hospital level and onset, Aboriginal women. [file 12884_2022_5222_MOESM5_ESM.tif]
